# Global Impacts of Simulating Organic Aerosol Phase State

**DOI:** 10.1021/acs.est.5c13238

**Published:** 2026-07-21

**Authors:** Yumin Li, Colette L. Heald, Ruqian Miao, Pedro Campuzano-Jost, Jose L. Jimenez

**Affiliations:** † Institute for Atmospheric and Climate Science, ETH Zurich, 8092 Zurich, Switzerland; ‡ State Key Laboratory of Regional Environment and Sustainability, College of Environmental Sciences and Engineering, Peking University, Beijing 100871, China; § Cooperative Institute for Research in Environmental Sciences and Department of Chemistry, 1877 University of Colorado, Boulder, Colorado 80309, United States

**Keywords:** organic aerosol, phase state, viscosity, heterogeneous reaction, gas-particle partitioning, reactive uptake, global model

## Abstract

Organic aerosol (OA) in the atmosphere exists in liquid, semisolid, or solid states, controlled by molecular properties and environmental conditions. However, regional and global models typically assume that OA exists exclusively in the liquid phase. We develop a source-specific OA phase-state model that enables key heterogeneous processes, including gas-particle partitioning, reactive uptake, and ice particle nucleation, to be explicitly phase-state-dependent. Our simulations show that globally more than 70% of surface OA exists in a semisolid state, consistent with observations (*r* = 0.9, NMB = −15%). OA viscosity is higher over continental midlatitude regions and increases with altitude, with little to no liquid phase above 2.5 km due to generally lower temperatures and relative humidity, compared to the surface. The phase state substantially slows gas-particle partitioning and uptake while enhancing OA removal at higher altitudes, where solid-state OA can act as ice-nucleating particles. Consequently, global annual mean surface OA concentrations decrease by 28%, while concentrations at 7 km increase by 8%, leading to a 36% reduction in the vertical OA concentration gradient, with a modest decrease in the global OA burden (2.64 *Tg* to 2.32 *T*g). Incorporating the OA phase state improves agreement with observed vertical OA profiles from both near-source (EMERGE-AS: *r* from 0.92 to 0.93 and NMB from 136% to 75%) and remote (ATom: *r* from 0.3 to 0.6 and NMB from 122% to 96%) aircraft campaigns.

## Graphical abstract

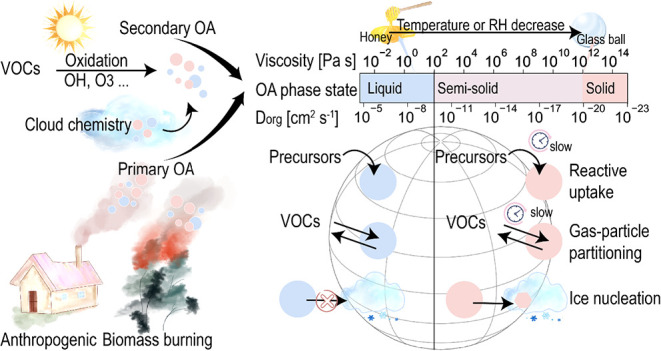

## 1 Introduction

Atmospheric organic aerosols (OA), which play a critical role in climate, public health, and biogeochemical systems, can be directly emitted as primary organic aerosols (POA) or formed through complex reactions as secondary organic aerosols (SOA).^1^ A key property governing OA behavior in the atmosphere is its phase state, ranging from liquid (viscosity <10^2^ Pa·s) to semisolid (10^2^–10^12^ Pa·s) and solid (>10^12^ Pa·s).^2,3^ Because OA viscosity is directly related to diffusional motion through the Stokes–Einstein relation,^2,4^ it influences the particle’s mixing time, growth, evaporation kinetics, gas uptake, water uptake, and CCN activation, thereby modulating heterogeneous chemistry and cloud microphysics.^2–6^ Laboratory and field measurements consistently show that OA viscosity increases with higher molar mass, larger O:C ratios, and reduced temperature and humidity, leading to the frequent occurrence of nonliquid OA under cold and dry atmospheric conditions.^2,3,7^ However, three-dimensional model simulations generally consider OA solely in the liquid state, thereby limiting accurate assessments of OA environmental and climatic impacts, especially in regions dominated by semisolid or solid OA.

Because high-viscosity OA undergoes fundamentally different chemical processing from liquid OA (TOC art), OA evolution should be described within a phase-state-dependent framework. Compared to liquid OA, semisolid and solid OA exhibit greatly reduced diffusion coefficients, leading to slower heterogeneous reactions through reactive uptake.^4,8–10^ This kinetic limitation suppresses SOA formation and slows OA aging, thereby altering OA lifetime, spatial distribution, and chemical composition. High particle viscosity slows gas-particle partitioning and suppresses the re-evaporation of semivolatile compounds once condensed,^11,12^ thereby enabling longer-range transport of particle mass. In addition, solid OA can act as ice-nucleating particles (INPs) through immersion freezing and deposition nucleation, potentially influencing glaciated cloud formation and precipitation.^3,13–17^ Although recent box, regional, and global modeling studies have begun to incorporate SOA phase states,^18–26^ to calculate the mixing equilibria time under high viscosity conditions,^5,23,27^ no study to date has explored how treating OA phase state impacts simulated global OA evolution and abundance.

In this study, we developed a comprehensive modeling framework to quantify the global impacts of OA phase states by explicitly integrating phase state dynamics into atmospheric OA evolution in the GEOS-Chem model. We evaluated the simulated OA phase states against observations at sites around the world. We quantify how phase-state-dependent processes modulate OA concentrations and compare our findings with aircraft OA measurements. We conclude by discussing and identifying the key uncertainties associated with our simulation of the phase state impacts.

## 2 Model and Data

### 2.1 GEOS-Chem Model

We develop a source-specific global OA phase state simulation and implement the effects of phase state on OA chemistry and deposition using the GEOS-Chem three-dimensional global chemical transport model (v14.3.0, 10.5281/zenodo.10640536).^28^ GEOS-Chem is driven by the Modern-Era Retrospective Analysis for Research and Application, Version 2 (MERRA-2) assimilated meteorological data product from the NASA Goddard Global Modeling and Assimilation Office (GMAO). All species in GEOS-Chem are treated as externally mixed. Our global simulations for 2017 feature a horizontal resolution of 2° × 2.5° and a vertical resolution of 47 hybrid-sigma layers. We spin up the model for one year prior to the start of the simulation and use chemical and transport timesteps of 20 and 10 min, respectively.^29^ Additional simulations are performed for 2016–2018 to match the time periods of airborne campaigns described in Section 2.4.

We use the complex OA scheme that incorporates semivolatile POA.^30–35^ We also include POA from marine sources.^36^ Freshly emitted hydrophobic POA is allowed to convert to hydrophilic POA during atmospheric aging (Text S1). Hydrophobic and hydrophilic POA from all sources are assigned OA:OC ratios of 1.4 and 2.1, respectively.^33^ SOA formation is represented using a Volatility Basis Set (VBS)^30^ framework for anthropogenic precursors and terpenes, while irreversible uptake schemes are applied to isoprene SOA,^31^ organic nitrate SOA,^34,35^ and glyoxal and methylglyoxal SOA.^37^ GEOS-Chem treats all SOA as hydrophilic with a molecular weight of 250 g mol^–1^; we adopt O:C ratios of 0.7 for biogenic SOA and 1.1 for anthropogenic SOA (Text S2).^38^

We use the Community Emissions Data System (CEDS) for anthropogenic emission,^39^ but supersede this by regional inventories where available (Text S1). We use the year-specific Global Fire Emissions Database (GFEDv4.1s) for biomass burning emissions^40^ and MEGAN (v2.1)^41^ for biogenic VOC emissions.

### 2.2 OA Phase State Module and Viscosity Parametrization

We implement an online phase state modeling framework in GEOS-Chem to calculate OA viscosity (η) and diagnose OA phase state. Due to limited direct η measurements, we predict η from source-specific glass transition temperatures (*T*_g_) using the Vogel–Fulcher–Tammann relationship (eq 1) following the approach of previous studies.^18,22,23,42^

1 $$\eta =\eta_{\infty }e^{T_{0}D_{f}/(T-T_{0})}$$

where η_∞_ = 10^–5^ Pa·s is the viscosity at infinite temperature, *D*_f_ is the fragility parameter (*D*_f_ = 10 for *T* ≤ *T*_g_;^22,23,42,43^ *D*_*f*_ = 30 for *T* > *T*_g_ ^19^), and *T*_0_ is the Vogel temperature derived from *T*_g_ as $T_{0}=\frac{39.17T_{g}}{D_{f}+39.17}$ . We neglect potential variations in *D*_f_ among OA species, which may introduce uncertainties of 1–3 orders of magnitude in viscosity predictions.^42^

For POA, we adopt dry *T*_g_ values of 231.0 K for hydrophobic POA^44,45^ and 260.5 K for hydrophilic POA,^46^ derived from bulk lab measurements of fresh biomass burning POA. The RH effect on *T*_g_ is also based on measurements: hydrophilic POA *T*_g_ decreases with increasing RH (0–100%), while hydrophobic POA remains largely unaffected until RH > 95%.^44,45^ These values are also applied to anthropogenic POA due to the lack of data (Text S2). Although we select relatively high literature values, we may underestimate actual viscosity given that aged biomass burning POA generally exhibits higher *T*_g_ and viscosity (Text S2).^45^

For SOA, we calculate *T*_g_ from the molecular weight and the O:C ratio, yielding dry *T*_g_ values of 297.9 K for biogenic SOA and 325.5 K for anthropogenic SOA.^18,42^ The RH effect on SOA *T*_g_ is parametrized with the Gordon–Taylor approach,^2^ consistent with previous work (Text S2).^18,23^ Although three distinct *T*_g_ prediction methodologies exist, their results are consistent.^18,42,43,47^ Our values fall within the *T*_g_ range for SOA (280–360 K) reported from other prediction methodologies.^43^ Our predicted anthropogenic SOA *T*_g_ exceeds biogenic SOA *T*_g_ by 30–50 K, also in agreement with laboratory measurements.^48,49^ The uncertainty in predicting *T*_g_ for dry SOA (±75 K) mainly reflects uncertainties in O:C and molecular weight assumption (Text S2).

Phase separation is typically observed between semisolid OA and inorganic aerosols, although its occurrence can depend on RH and composition.^3,50–53^ Here, we assume external mixing between OA species, neglecting interactions between them and the effect of inorganic components on *T*_g_. We calculate the *T*_g_ of total OA as a mass-weighted mean across only POA and SOA species (eq 2)­

2 $$T_{g,total}=\frac{\sum m_{OA,i}\times T_{g,i}}{m_{TOA}}$$

where *T*_g,total_ is the *T*_g_ of total OA, m_OA,*i*_ is the dry mass of each OA species, and *T*_g,*i*_ is the *T*_g_ of each OA species as calculated above. *i* indicate: hydrophobic POA, hydrophilic POA biogenic SOA, and anthropogenic SOA. *m*_TOA_ is the total mass concentration of all OA species. When hygroscopic growth-adjusted OA mass is used for mass-weighting instead, the resulting change in *T*_g,total_ is minor (typical <1 K; Text S2).

The overall uncertainty in source-specific OA *T*_g_ consequently translates into uncertainties in viscosity predictions and the corresponding simulation of heterogeneous chemical processes. Our sensitivity simulation shows that a 50 K shift in *T*_g_ alters viscosity by only 1–2 orders of magnitude for liquid or low-viscosity semisolid OA, with larger impacts in the high-viscosity semisolid regime (Text S2, Figure S1). However, once OA transitions into the solid state, chemical reactivity is effectively negligible, rendering it largely insensitive to further variations in the viscosity.

### 2.3 Phase State-Dependent OA Reactions

We use our predicted source-specific OA viscosity (η_*i*_) to calculate the diffusion coefficient of each OA species (*D*_org,*i*_, eq 3). This approach allows us to account for source-specific OA viscosity when updating reaction rates, directly linking the OA phase state of each source to its own reaction kinetics.^2–4^

3 $$D_{\mathrm{org},i}=\frac{k_{B}T}{6\pi {\eta_{i}r}_{\mathrm{eff}}}$$

where *k*_B_ is the Boltzmann constant (J K^–1^), *T* is the temperature (K), *r*_eff_ is the effective hydrodynamic radius of OA, assumed to be 1 nm following previous studies.^2,25^ Some studies have also adopted smaller values of *r*_eff_,^20^ which would result in a larger *D*_org_ value under the same viscosity. Overall, *D*_org_ uncertainty is within a factor of 10 for a given viscosity based on previous studies.^2,4,7^ *i* include the hydrophobic POA, hydrophilic POA, biogenic SOA and anthropogenic SOA.

#### 2.3.1 Phase State-Dependent Reactive Uptake

We update the source-specific OA reactive uptake process based on their own *D*_org_. In the standard GEOS-Chem model, heterogeneous reaction rates are calculated using a reactive uptake coefficient (γ) that is either assumed as constant or estimated from resistance models assuming a uniform liquid aerosol surface.^31,34,54^ Considering the tendency of high viscosity OA to exist in a phase-separated state,^3,50–52^ we update γ for semisolid and solid states by assuming a core–shell structure with a thin high-viscosity shell (1/10 of particle radius) surrounding a liquid core in our standard simulation rather than a homogeneous morphology. We also conduct sensitivity simulations with a thicker shell and fully mixed homogeneous morphology assumptions (Text S3). When the shell is relatively thin, the responses of the phase state are similar for both homogeneous mixing and core–shell assumptions. When a thicker shell is assumed, the reduction of γ occurs earlier than the liquid-to-semisolid transition. The uncertainty associated with OA thickness produces a several-fold variability of γ (Text S3.1). The revised uptake coefficient (eq 4) is applied to isoprene SOA, organic nitrate SOA, glyoxal, and methylglyoxal SOA formation.

4 $$\frac{1}{\gamma }=\frac{1}{\gamma_{0}}+\frac{\omega l_{\mathrm{org}}}{4\mathrm{RT}H_{\mathrm{org}}^{*}D_{\mathrm{org}}}\;\frac{r}{r_{c}}$$

where γ_0_ is the reactive uptake coefficient in liquid phase, *l*_org_ is the shell thickness (cm), *r* is the particle radius (1.0 × 10^–5^ cm), and *r*_c_ is the core radius (cm), *H*_org_^*^ is the effective Henry’s Law constant (M atm^–1^), R is the universal gas constant.

#### 2.3.2 Phase State-Dependent Gas-Particle Partitioning

We apply source-specific OA *D*_org_ to slow gas-particle partitioning in GEOS-Chem for viscous particles. In the standard VBS scheme applied in 3D models, including GEOS-Chem, gas-particle organic partitioning is treated as absorptive equilibrium partitioning dependent on the temperature-dependent volatility (C*) and the available absorbing organic mass within each chemical timestep (20 min for our simulations).^30,55^ This equilibrium assumption is appropriate for much of the atmosphere, but for low volatility and high viscosity organics, the time scale to achieve equilibrium likely exceeds this time step (Text S3.2). Typically, high viscosity OA with low bulk diffusivity can substantially slow particle-phase mixing.^56^ To represent the phase state limitation, we calculate the particle mixing time scale using eq 5 ^5^ and use it to impose an additional kinetic constraint on the approach to the VBS predicted equilibrium state. Specifically, the particle concentration is relaxed toward the VBS predicted equilibrium state according to eq 6.

5 $$\tau_{\mathrm{mix}}=\frac{r^{2}}{\pi^{2}D_{\mathrm{org}}}$$

6 $$C_{p}\left(t+\Delta t\right)=\{\begin{array}{ll} C_{p,eq}^{VBS} & \tau_{mix}\leq \Delta t\\ C_{p}\left(t\right)+\left[C_{p,eq}^{VBS}-C_{p}\left(t\right)\right]\left(1-\exp^{-\Delta t/\tau_{p}}\right) & \tau_{mix}>\Delta t \end{array}$$

where *C*_p,eq_^VBS^ is the equilibrium particle concentration predicted by the VBS scheme diagnosed by the VBS framework from temperature-dependent volatility and the available absorbing organic mass, Δ*t* is the chemical time step (20 min), and τ_p_ is the phase state delay time scale. When τ_mix_ > Δ*t*, we set τ_p_ = τ_mix_ to represent diffusion-limited time scale adjustment; otherwise, the default treatment is retained.

We note that our scheme does not address the underlying limitations of the initial VBS equilibrium partitioning assumption beyond viscosity and thus does not represent the full atmospheric equilibration time scale, which also depends on volatility, OA mass concentration, gas-phase diffusion, and interfacial mass accommodation.^56^ These factors and the conditions where they are prevalent and could contribute to biases in simulated OA are discussed in Text S3.2 and Section 4. This underlying bias is present in the baseline VBS scheme and with the addition of our phase-dependent scheme; thus, relative differences capture the phase-only effects.

#### 2.3.3 Phase State-Dependent Wet Deposition

In the standard GEOS-Chem wet deposition scheme, OA are not considered ice-nucleating particles (INPs) between −20 and −40 °C. However, highly viscous semisolid or solid OA, which can act as cloud condensation nuclei, may also serve as INPs at temperatures below −20 °C (253.15 K).^13–17^ Furthermore, at temperatures below −40 °C (233.15 K),^13,14,16^ all OA may act as potential INPs. We therefore revised the wet deposition scheme to allow OA species with viscosity greater than 10^12^ Pa·s (solid state) to act as ice nuclei and be removed once incorporated into clouds when the temperature is below −20 °C. This treatment is conservative, as the highest-viscosity semisolid OA may also retain some potential for INPs.^3,13,14^

### 2.4 Aircraft Observation Data

We used OA concentration measurements from two aircraft campaigns representing distinct environments:

(1) near-source regions from the Effect of Megacities on the Regional to Global Scales-East Asia (EMERGE-AS) campaign in spring 2018, with 1 min OA data from C-ToF-AMS^57,58^

(2) Remote marine environments from the Atmospheric Tomography (ATom) 1–4 campaigns across four seasons (2016–2018), with 1 min OA data from HR-ToF-AMS.^59^ To focus on aged marine air, we used ATom data from the Pacific and Antarctic Oceans.

We preprocess the observational data as follows to compare with our simulation: (1) remove concentrations above the 97th percentile to filter out localized plumes not resolved by the model;^60^ (2) regrid to the GEOS-Chem 2° × 2.5° resolution; (3) stratify from the surface to 10 km in 500m bins. Statistical performance is quantified using the Pearson correlation coefficient $\left(r_{\mathrm{xy}}=\frac{\sum_{i=1}^{n}\left(x_{i}-\mathrm{x̅}\right)(y_{i}-\mathrm{y̅})}{\sqrt{\sum_{i=1}^{n}{\left(x_{i}-\mathrm{x̅}\right)}^{2}}\times \sqrt{\sum_{i=1}^{n}{\left(y_{i}-\mathrm{y̅}\right)}^{2}}}\right)$ and normalized mean bias $\left(\mathrm{NMB}\;\%=\left[\frac{\sum_{i=1}^{n}\left(y_{i}-x_{i}\right)}{\sum_{i=1}^{n}x_{i}}\right]\times 100\%\right)$ .

## 3 Results

### 3.1 Global Simulated OA Phase State

Figure 1 illustrates the simulated OA phase states from different sources in 2017, and the mass concentration-weighted average total OA phase state distribution. A clear vertical gradient is apparent: OA is mostly liquid or semiliquid below 2.5 km but becomes predominantly semisolid or solid above. Additionally, hydrophobic POA exhibits higher viscosity at high latitudes and lower viscosity in the tropics, consistent with the temperature dependence of its phase state. In contrast, the viscosity of hydrophilic POA, biogenic SOA, and anthropogenic SOA is strongly affected by both temperature and RH, leading to higher viscosity not only in polar regions relative to the tropics (due to lower temperatures) but also in midlatitude continental regions where RH is reduced. Furthermore, anthropogenic SOA is generally more viscous than biogenic SOA and hydrophilic POA, due to its relatively higher O:C ratio.^3,38^

**Figure 1:**
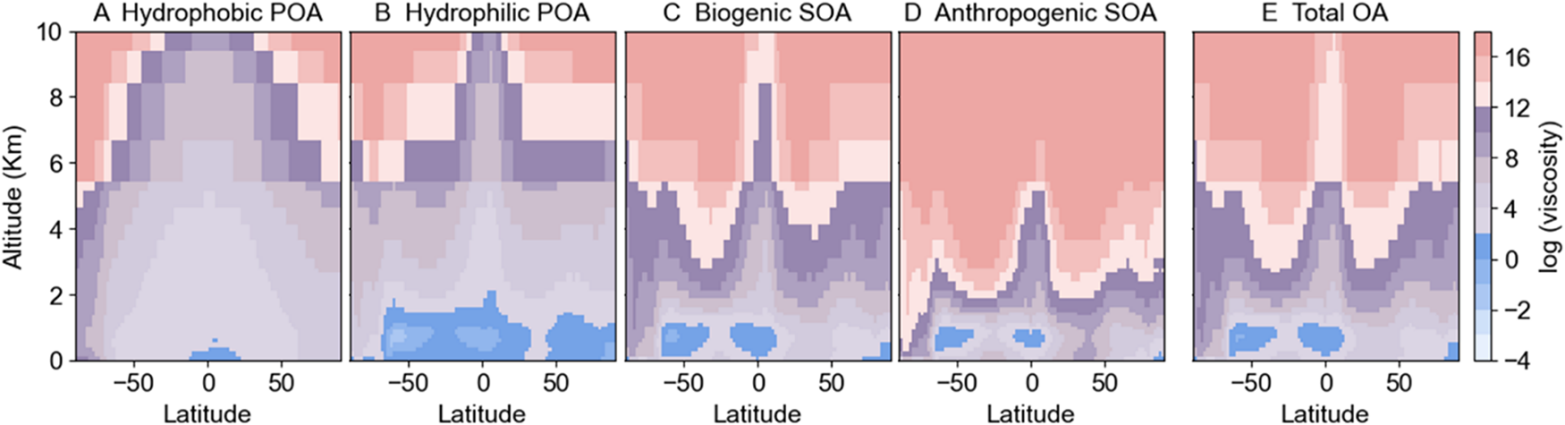
Simulated meridional mean source-specific OA and total OA viscosity. (A) hydrophilic POA, (B) hydrophobic POA, (C) biogenic SOA, (D) anthropogenic SOA, (E) total OA.

To evaluate our explicitly simulated global OA phase state, we compare the model against surface observations from 25 sites globally (Figure 2). Observations include direct phase state measurements using the particle bounce method and viscosity estimates derived from volatility distributions from previous studies.^23,43,61,62^ The particle bounce method distinguishes only liquid or nonliquid states, whereas volatility-based analyses provide quantitative viscosity estimates. The observed OA phase states around the world range from liquid to solid states, with a predominant distribution in the semisolid state (Figure 2A, purple). Our simulation is consistent with the particle bounce observations, except at Seosan, Korea, where higher RH from local marine influence likely reduces viscosity but is not fully captured by our grid resolution (Figure 2B). In addition, our simulated viscosity correlates strongly with observations from volatility distribution measurements (Figure 2C; *r* = 0.9, NMB = −15%); a slight underestimation likely stems from not accounting for more viscous aged POA in our simulation.^45^ Our high-end and low-end viscosity sensitivity simulations (SI Text S4) are shown as orange uncertainty bars (along the *y*-axis, Figures 2B,2C). The high-end assumption overestimates the observed viscosity (NMB = 53%), whereas the low-end assumption underestimates it (NMB = −58%). The lack of observational data limits our ability to evaluate simulated viscosity at higher altitudes. The spatial distribution of simulated OA viscosity aloft closely resembles the surface conditions (Figure S2).

**Figure 2:**
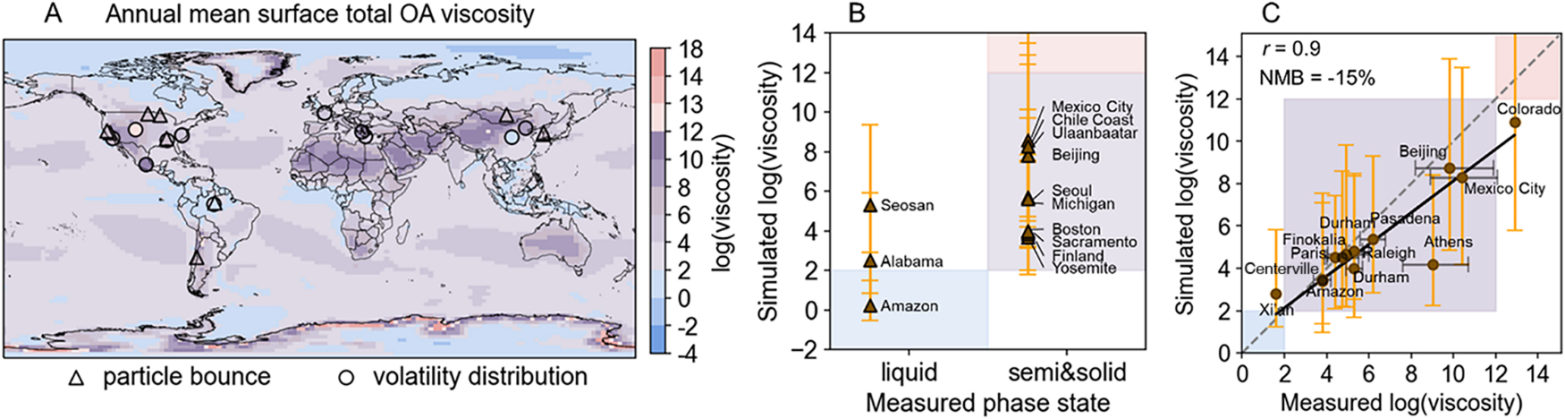
Observed (from 2006 to 2019) and simulated (2017) global annual mean surface total OA viscosity. Liquid phase in blue, semisolid in purple, and solid in pink (A). Observed (symbols, triangles represent the locations of particle bounce measurements, circles represent volatility distribution measurements) and simulated (filled contours) annual mean surface total OA viscosity. (B) Observed OA phase state from particle bounce measurements compared with simulated OA viscosity. (C) Scatter plot of simulated viscosity against viscosity measured from volatility distribution. The slopes (s) of the reduced major-axis regression lines (solid lines) and the Pearson correlations (*r*) are inset. The gray dashed line represents the 1:1 line. The OA viscosity at the Colorado Rocky Mountains site on the scatter plot corresponds to simulation and measurement at an altitude of 2–2.5 km. The orange uncertainty bars (along the *y*-axis) indicate the simulated viscosity uncertainty range from the high-end and low-end sensitivity simulations (SI Text S4), while the black uncertainty bars (along the *x*-axis) represent the range of the observed viscosity.^43^

Our simulation suggests that the surface global spatial distribution of the total OA phase state (Figure 2A) is mainly controlled by RH (Figure S3). The highest viscosity is simulated in arid and semiarid continental regions (e.g., southwestern US, southern Africa, Sahara, Arabian Peninsula, northwestern China, Australia). While marine regions exhibit lower viscosity than terrestrial areas, midlatitude marine surface OA is still predominantly semisolid, though observational constraints are lacking. This spatial distribution in our total OA viscosity simulation aligns with previous global and regional SOA-only viscosity simulations,^18,21–23^ as RH is also a key factor influencing the SOA phase state. However, our explicit treatment of both POA and SOA allows us to evaluate the total OA viscosity. Compared with SOA viscosity reported in previous studies,^18,21–23^ our simulated total OA viscosity is around 10–10^2^ times higher in low-viscosity regimes but 10–10^2^ times lower in high-viscosity regimes, reflecting the fact that SOA exhibits higher viscosity under dry conditions and a stronger hygroscopic response to RH than POA (Section 2.2). Key differences occur in phase transition regions: (1) total OA remains in the upper semisolid range (e.g., southwestern US, northwestern China, Sahara) where SOA is solid; (2) total OA resides in the lowest semisolid range (e.g., remote high latitude ocean) where SOA is liquid. These distinctions strengthen the correlation between our simulated and observed viscosities compared with previous studies.^18,21–23^

### 3.2 Impact of Global OA Phase State on Reactive Uptake

Figure 3A­(1–3) shows the global distribution of simulated OA concentration differences between scenarios with and without phase state effects during reactive uptake, which controls the formation of isoprene SOA, glyoxal/methylglyoxal SOA, and organic nitrate SOA. Incorporating phase state reduces the global SOA burden by 0.39 *Tg* (33%), leading to a 28% decrease in mean surface total OA concentration (locally range 0.6–60%), while the reduction at 7 km averages 14% (locally ranges 3–36%) (Figures 3 and 4). This decrease reflects slower reactive uptake in semisolid or solid OA than in liquid OA, which reduces SOA production at the surface and its transport to remote and high-altitude regions. Spatially, SOA reductions align closely with the distribution of high viscosity regions (Figures 2 and S2), with larger decreases in high viscosity areas and smaller reductions in low-viscosity regions. The impact on total OA concentration is more pronounced in the Southern Hemisphere, reflecting the larger contribution of isoprene SOA to total OA there (Figure S4).

**Figure 3:**
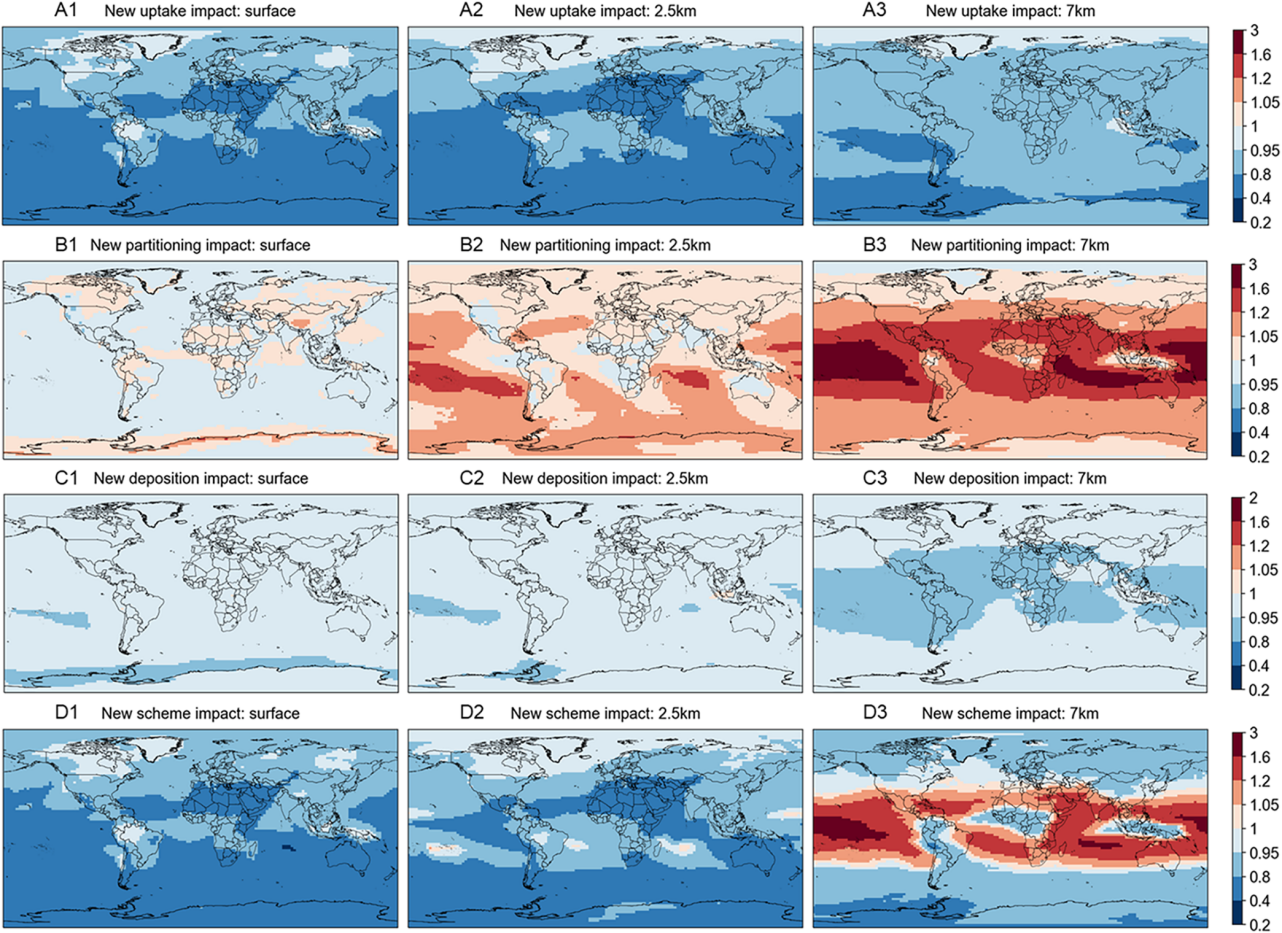
Phase state impact of global simulated annual mean OA concentrations at the surface, 2.5 km, and 7 km altitudes. (A1–A3) Phase state-dependent reactive uptake impact; (B1–B3) phase state-dependent gas-particle partitioning impact; (C1–C3) phase state-dependent deposition impact; (D1–D4) overall phase state-dependent scheme. The impact is indicated by the ratio between with and without considering the phase state in each scheme.

**Figure 4:**
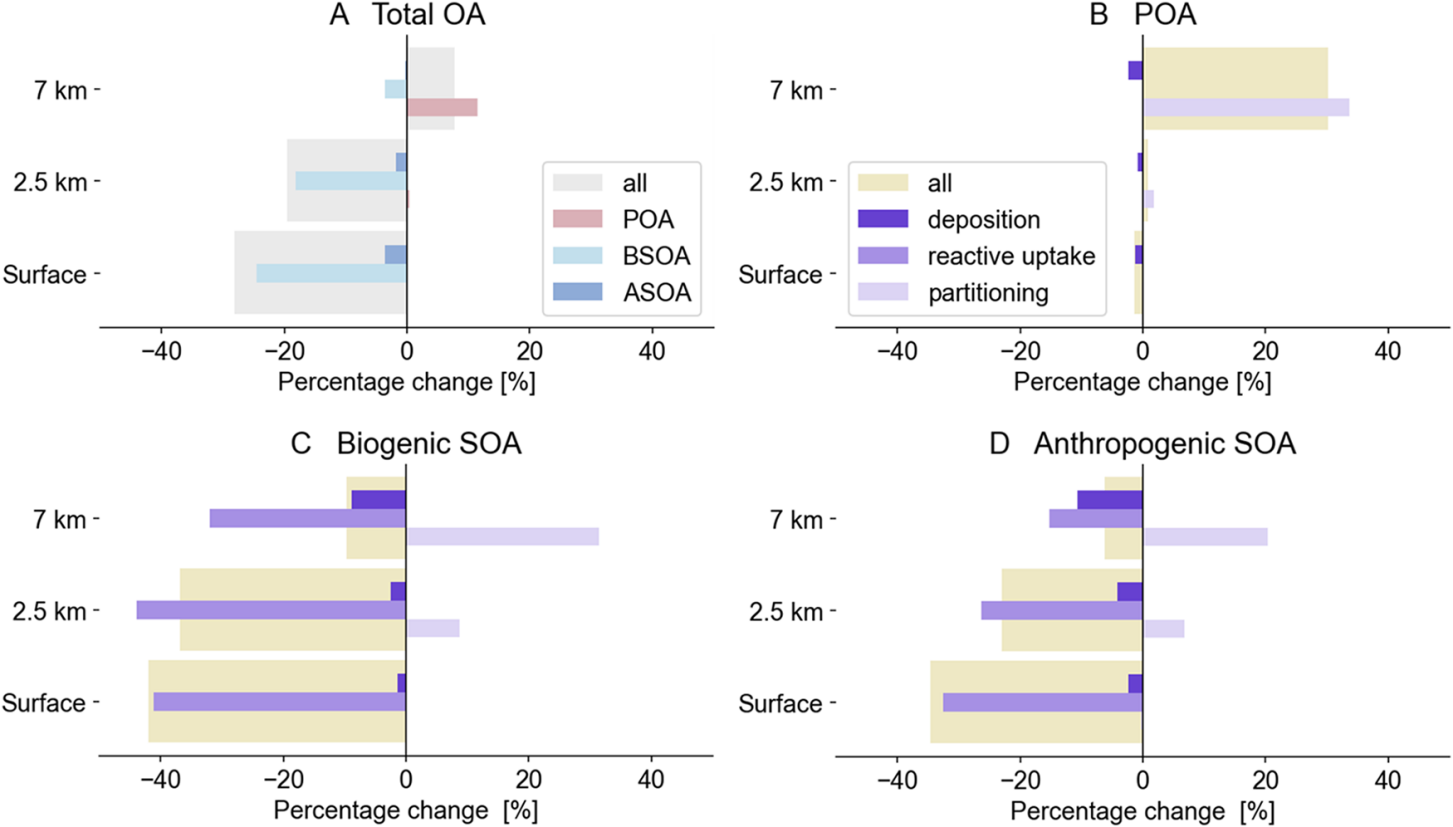
Simulated global mean OA concentration changes due to phase state effects at different altitudes (surface, 2.5 km, and 7 km). (A) total OA (B) POA, (C) biogenic SOA, (D) anthropogenic SOA.

### 3.3 Impact of Global OA Phase State on Gas-Particle Partitioning

Figure 3B (1–3) illustrates the global distribution of simulated OA concentration differences between scenarios that account for the impact of phase state during gas-particle partitioning and those that do not. Gas-particle partitioning governs the formation of semivolatile POA and all SOA except those derived from isoprene, organic nitrates, and glyoxal/methylglyoxal. When the impact of OA phase state is considered, the global OA burden increases from 2.64 *Tg* to 2.74 *Tg*, with SOA and POA contributions increasing by 0.07 *Tg* and 0.03 *T*g, respectively. The increase in SOA concentration is greater than the POA concentration due to the higher relative viscosity of SOA compared to POA (Figure 1).

At the surface, the average global OA concentration increases only slightly by 0.06%, but spatial variations are substantial, ranging from local decreases of 9% to local increases of 26%. In near-source and high-viscosity regions such as the southwestern United States, India, and Australia, OA concentrations decrease because higher viscosity restricts gas to particle partitioning, limiting OA formation. This limitation potentially allows more gas-phase organics to be transported away from sources, particularly to regions with viscosities lower than those of the source regions, such as northern North America and southern Australia, where OA concentrations increase. In remote and high-viscosity areas, including the Antarctic, northern Africa, and northwestern China, high viscosity also limits particle-to-gas transitions, further enhancing OA concentrations.

With increasing altitude, OA viscosity increases, further hindering particle partitioning back to the gas phase and prolonging OA lifetime. At 7 km, where OA predominantly exists in the solid state, global average OA concentrations increase by 28% (Figure 3). In remote low-latitude ocean regions, OA concentration enhancements can exceed 130% due to the highest viscosity at these altitudes (Figure S2).

### 3.4 Impact of Global OA Phase State on Deposition

Figure 3C (1–3) illustrates the global distribution of the simulated OA concentration ratio between scenarios that consider the impact of phase state during deposition and those that do not. Incorporating solid-state OA, which can participate in immersion freezing and deposition nucleation, leads to a slight decrease in OA concentrations from the surface to higher altitudes. The total OA burden decreases by 0.03 *Tg*, with 0.01 *Tg* of POA and 0.02 *Tg* of SOA.

At the surface, the mean OA concentration decreases by only 1%, reflecting both the low proportion of solid OA and the limited cloud scavenging under typical surface conditions, as supercooled clouds are rare except in extreme polar regions. At higher altitudes, cooler temperatures increase the fraction of solid OA and enhance immersion freezing, resulting in an average 5% reduction at 7 km with local decreases ranging from 1 to 19%. This reduction reflects the spatial distribution of OA viscosity and clouds: as more OA exists in the solid state with cool clouds, the effect of phase-state-dependent deposition becomes more pronounced. Notably, our simulation represents a conservative estimate, as the highest-viscosity semisolid OA may also participate in immersion freezing.^3,13,14^

### 3.5 Overall Phase State Impact on Global OA

Figure 3D (1–3) summarizes the net impacts of the phase state on the global distribution of OA concentrations, while Figure 4A illustrates the average changes in the global vertical gradient and source-specific OA contributions. Globally, accounting for phase state decreases mean OA surface concentrations by 28%, decreases concentrations at 2.5 km by 19%, and increases concentrations at 7 km by 8%, resulting in an approximately 36% attenuation of the mean vertical OA gradient. The total OA burden decreases from 2.64 *Tg* to 2.32 *Tg*, with SOA decreasing from 1.19 *Tg* to 0.85 *Tg* and POA increasing slightly from 1.45 *Tg* to 1.48 *Tg*. Sensitivity simulations using high-end and low-end viscosity assumptions show correspondingly stronger and weaker phase-state impacts (Text S4, Figure S5), but in all cases, accounting for phase state consistently reduces the vertical gradient in OA concentrations.

At the surface, the main effect of increased viscosity is reduced reactive uptake, which lowers SOA production. With increasing altitude, the influence of slower reactive uptake diminishes, while the effects of inhibited gas-particle partitioning and enhanced wet deposition become more pronounced. At 2.5 km, phase-state-dependent partitioning increases both SOA and POA concentrations in midlatitude oceans and along the Antarctic coast, partially offsetting reductions from decreased reactive uptake. By 7 km, slower gas-particle partitioning dominates, leading to overall OA increases, particularly over remote midlatitude oceans, while concentrations in other regions remain slightly lower. Overall, enhanced deposition of solid OA contributes a smaller effect on global OA concentrations than phase-state-dependent reactive uptake and gas-particle partitioning, although this outcome may depend on the model representation of deposition and cloud scavenging processes.

Figure 4B–D further shows distinct global average vertical responses of POA and SOA to phase state. SOA concentrations decrease mainly at the surface and show slight decreases at higher altitudes, whereas POA exhibits the opposite pattern with increases at high altitudes and minimal changes or slight decreases near the surface. The phase state affects SOA more than POA due to (1) higher SOA viscosity relative to POA, (2) reactive uptake processes that only govern SOA formation, and (3) SOA concentration changes occurring mainly at the surface due to reactive uptake, whereas POA modifications manifest mainly at higher altitudes via gas-particle partitioning.

### 3.6 Evaluation of Phase State-Dependent Simulated OA against Aircraft Observation Data

As discussed above, considering the phase state decreases the vertical OA concentration gradient. To assess the impact of phase state on the vertical OA distribution, we compared model simulations with and without phase state against aircraft observations from two regions: near-source East Asia during the 2018 EMERGE-AS campaign (Figure 5A1–A2, Figure S6A) and the remote Pacific Ocean and Antarctic coast during ATom 2016–2018 (Figure 5B1–B2, Figure S6B). Observations are based on direct AMS measurements (black lines).

**Figure 5:**
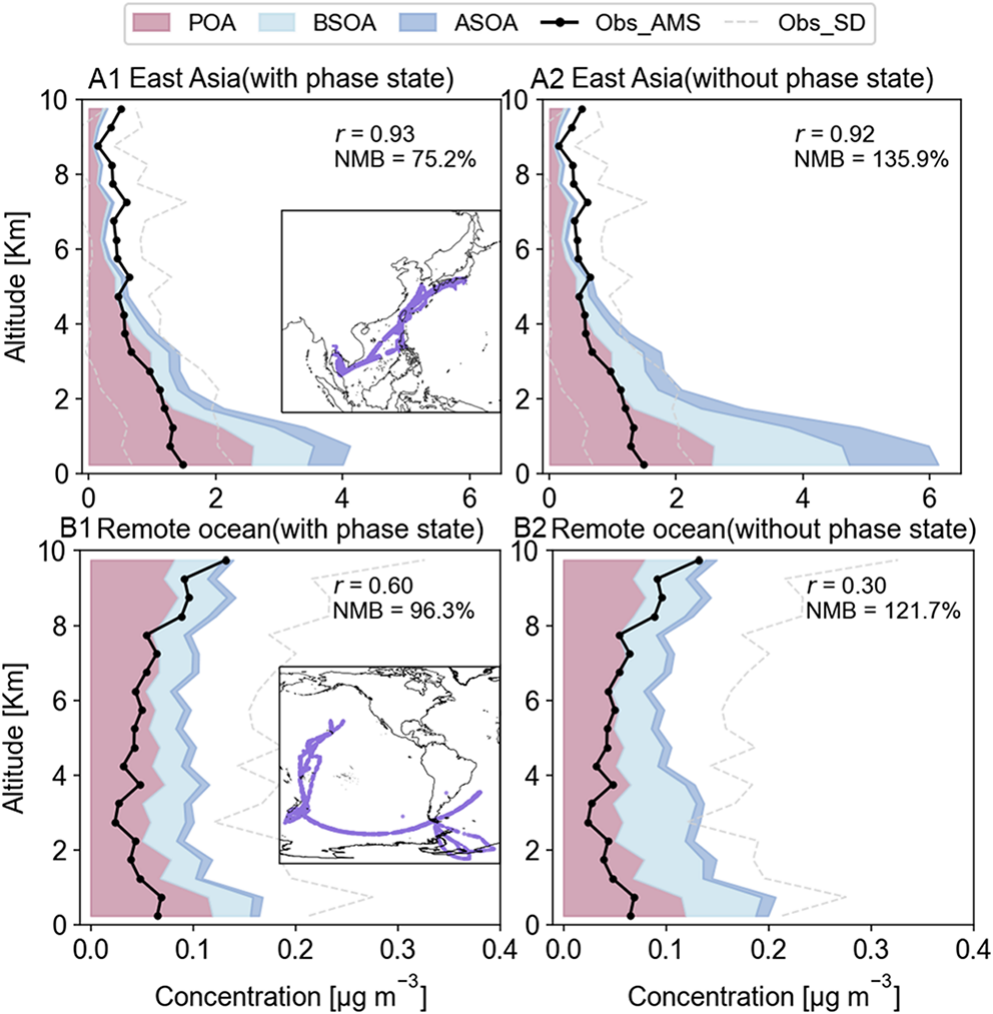
Comparison of simulated mean OA vertical concentration profiles with and without phase state treatments against aircraft measurement data. Simulated OA concentration profiles with (A1) and without (A2) phase state vs measurements from the EMERGE-AS campaign in East Aisa; Simulated OA concentration profiles with (B1) and without (B2) phase state vs measurements from ATom1 to ATom4 campaign in remote ocean areas. The Pearson correlation coefficients (*r*) and normalized mean bias (NMB) are displayed in the inset. Obs_AMS indicates the AMS measured concentrations and the Obs_SD indicates the standard deviation of the observations in each altitude bin. The aircraft flight tracks are also shown in an inset.

In both regions, incorporating the phase state improves the agreement with observed vertical OA gradients (Figure 5, Figure S6). Over in East Asia, observations show a strong surface-to-high-altitude decrease. Both simulations correlate well with measurements (*r* increases from 0.92 to 0.93), but accounting for phase state reduces the overestimation of near-surface OA, decreasing the NMB from 136% to 75% due to lower SOA concentrations, yielding a more realistic vertical trend. At higher altitudes, reductions in SOA and increases in POA partially offset each other, allowing both simulations to capture the OA levels. Over the remote Pacific and Antarctic coast, observed OA concentrations decrease from the surface to midtroposphere before rising at higher altitudes. The phase-state-dependent simulation slightly lowers near-surface OA (both POA and SOA), while at higher altitudes, the increase in POA and decrease in SOA balance, maintaining almost unchanged total OA, but with a higher POA fraction. This adjustment clearly improves the correlation (*r* from 0.30 to 0.60) and reduces NMB from 122% to 96%.

We note that atmospheric OA abundance is controlled by many factors, and differences between model and observations may reflect biases unrelated to the phase state. In addition, AMS measurements with a PM_1_ inlet may systematically underestimate fine OA (PM_2.5_),^63,64^ introducing further uncertainty (Text S5, Figure S7).^65,66^ Thus, this evaluation is not quantitatively definitive. Nonetheless, these results demonstrate that incorporating the phase state substantially improves the representation of vertical OA gradients and moderately enhances overall model performance.

## 4 Discussion

Our work introduces a phase-state-dependent OA simulation framework within a global three-dimensional model and, for the first time, explores how the phase state influences the atmospheric evolution of OA concentrations. Our simulations reveal that OA from different sources responds differently to environmental conditions when the phase state is considered, leading to notable changes in vertical profiles and source contributions. These variations have potential implications for the climate and environmental effects of OA. For instance, OA concentrations increase at high altitudes, where aerosols can more efficiently absorb solar radiation, potentially modifying the radiative forcing. High-viscosity OA also facilitates long-range transport of toxic pollutants in the particle phase, with implications for human health risk assessments.^67,68^

The predictive capability of our phase-state-dependent framework relies on limited laboratory and field constraints, as well as parametrized values (sections 2.2 and 2.3). Semisolid OA represents the most critical regime requiring careful consideration of uncertainty: near-surface OA is dominated by this state, yet variations in viscosity within this range can alter chemical reaction rates by orders of magnitude. The main uncertainties arise from two aspects: (1) phase-state prediction and (2) viscosity-dependent reactions.

Phase state predictions are sensitive to factors such as OA aging, aerosol mixing state, particle morphology, and source-dependent chemical composition, often approximated using bulk assumptions. For example, hydrophobic OA may phase separate from hydrophilic OA^69^ with different viscosities, and particles can exhibit diverse morphologies that cannot be fully captured by an idealized core–shell representation. However, comparison with observed viscosity (Figure 2) suggests that our scheme captures the phase state without systematic bias. Additional viscosity measurements are nevertheless needed to further evaluate and refine the parametrization.

Uncertainty in viscosity-dependent reactions is also influenced by simplifying assumptions regarding particle size, morphology, and hygroscopicity, whose broader applicability remains poorly constrained by observations as well as by limitations in the underlying model framework. For gas-particle partitioning, our phase state treatment improves the representation of high-viscosity OA compared with the standard VBS-only scheme, although biases likely remain for a subset of low-volatility organics, based on estimated condensation and evaporation time scales from Li and Shiraiwa.^56^ For low volatility species under high viscosity conditions, the phase-state-dependent scheme may overestimate condensation times and thus introduce a low bias in OA concentrations; however, this regime accounts for only about 2% of total OA mass in our simulations (Text S3.2). For low-volatility species under low-viscosity conditions, the standard VBS scheme still tends to underestimate evaporation times. This limitation does not affect the general assessment of phase state impacts based on the comparison between the two schemes. However, because some low-volatility species that were previously treated by the standard VBS scheme are instead treated by the phase-state-dependent scheme, part of the difference between the two simulations reflects correction of the VBS bias rather than the effect of phase state alone. As a result, the contribution of the phase state may be slightly overestimated here, although this effect involves only about 3% of OA mass (Text S3.2). Absorbing organic mass can also introduce several-fold uncertainties for equilibration time for low volatility organics,^12,56^ but its influence is smaller than that of volatility and viscosity (Text S3.2). Further improvement in gas-particle partitioning would require a complex treatment that distinguishes condensation from evaporation and resolves their different kinetic controls. Additional uncertainty arises from model representations of cloud scavenging and wet deposition when assessing phase state impacts on global OA burden. Moreover, as a global-scale model, GEOS-Chem cannot capture highly polluted urban regions, where mixing with inorganic salts may be important.^70^

Overall, our results underscore that the assumption of uniformly liquid OA in a three-dimensional model is inadequate. This work represents an initial step toward quantifying the influence of the phase state on global OA abundance. To advance this framework, targeted laboratory studies are essential, particularly to quantify the viscosity of OA from different sources, viscosity evolution during aging, and to measure viscosity-dependent heterogeneous reaction rates. Such data would improve parametrizations linking chemical kinetics to viscosity dynamics.

Concurrently, field measurements of OA viscosity, especially in remote and high-altitude environments, are critical for validating and constraining global model predictions. Collectively, these improvements will enable more robust simulations to assess the climatic and environmental impacts of OA in the future.

## Supplementary Material



## Data Availability

The GEOS-Chem model code for phase state simulation, the model configuration files, and the data underlying the figures included in this manuscript are available at https://zenodo.org/records/21264967.

## Notes

Details of the GEOS-Chem model, organic aerosol and phase state simulations, and results of sensitivity simulations (PDF)

## Abbreviations

OA: organic aerosol
POA: primary organic aerosol
SOA: secondary organic aerosol
INPs: ice-nucleating particles

## References

[1] Forster, P. ; Storelvmo, T. ; Armour, K. ; Collins, W. ; Dufresne, J. L. ; Frame, D. ; Lunt, D. J. ; Mauritsen, T. ; Palmer, M. D. ; Watanabe, M. ; Wild, M. ; Zhang, H. The Earth’s energy budget, climate feedbacks, and climate sensitivity. In Climate Change 2021: The Physical Science Basis. Contribution of Working Group I to the Sixth Assessment Report of the Intergovernmental Panel on Climate Change; Masson-Delmotte, V. ; Zhai, P. ; Pirani, A. ; Connors, S. L. ; Péan, C. ; Berger, S. ; Caud, N. ; Chen, Y. ; Goldfarb, L. ; Gomis, M. I. ; Huang, M. ; Leitzell, K. ; Lonnoy, E. ; Matthews, J. B. R. ; Maycock, T. K. ; Waterfield, T. ; Yelekçi, O. ; Yu, R. ; Zhou, B. , Eds.; Cambridge University Press, 2021; pp 923–1054.

[2] Koop T., Bookhold J., Shiraiwa M., Pöschl U. (2011). Glass transition
and phase state of organic compounds: dependency on molecular properties
and implications for secondary organic aerosols in the atmosphere. Phys. Chem. Chem. Phys..

[3] Reid J. P., Bertram A. K., Topping D. O., Laskin A., Martin S. T., Petters M. D., Pope F. D., Rovelli G. (2018). The viscosity of atmospherically
relevant organic particles. Nat. Commun..

[4] Shiraiwa M., Ammann M., Koop T., Pöschl U. (2011). Gas uptake
and chemical aging of semisolid organic aerosol particles. Proc. Natl. Acad. Sci. U.S.A..

[5] Shiraiwa M., Zuend A., Bertram A. K., Seinfeld J. H. (2013). Gas-particle partitioning
of atmospheric aerosols: interplay of physical state, non-ideal mixing
and morphology. Phys. Chem. Chem. Phys..

[6] Shrivastava M., Cappa C., Fan J., Goldstein A., Guenther A., Jimenez J., Kuang C., Laskin A., Martin S., Ng N., Petaja T., Pierce J., Rasch P., Roldin P., Seinfeld J., Shilling J., Smith J., Thornton J., Volkamer R., Wang J., Worsnop D., Zaveri R., Zelenyuk A., Zhang Q. (2017). Recent advances
in understanding secondary organic aerosol: Implications for global
climate forcing. Rev. Geophys..

[7] Gou, Y. ; Xie, M. ; Chen, J. The phase state and viscosity of organic aerosol and related impacts on atmospheric physicochemical processes: A review. Atmos. Environ. 2025, 343. 120985 10.1016/j.atmosenv.2024.120985.

[8] Slade J. H., Knopf D. A. (2014). Multiphase OH oxidation kinetics
of organic aerosol:
The role of particle phase state and relative humidity. Geophys. Res. Lett..

[9] Arangio A. M., Slade J. H., Berkemeier T., Poschl U., Knopf D. A., Shiraiwa M. (2015). Multiphase chemical
kinetics of OH radical uptake by
molecular organic markers of biomass burning aerosols: humidity and
temperature dependence, surface reaction, and bulk diffusion. J. Phys. Chem. A.

[10] Li J., Knopf D. A. (2021). Representation of
Multiphase OH Oxidation of Amorphous
Organic Aerosol for Tropospheric Conditions. Environ. Sci. Technol..

[11] Preston T. C., Zuend A. (2022). Equilibration times
in viscous and viscoelastic aerosol particles. Environ. Sci.-Atmos..

[12] Shiraiwa, M. ; Seinfeld, J. H. Equilibration timescale of atmospheric secondary organic aerosol partitioning. Geophys. Res. Lett. 2012, 39. 10.1029/2012GL054008.

[13] Wang, B. ; Lambe, A. ; Massoli, P. ; Onasch, T. ; Davidovits, P. ; Worsnop, D. ; Knopf, D. The deposition ice nucleation and immersion freezing potential of amorphous secondary organic aerosol: Pathways for ice and mixed-phase cloud formation J. Geophys. Res.: Atmos. 2012; Vol. 117 10.1029/2012JD018063.

[14] Kanji, Z. ; Ladino, L. ; Wex, H. ; Boose, Y. ; Burkert-Kohn, M. ; Cziczo, D. ; Krämer, M. Overview of Ice Nucleating Particles. In Ice formation and evolution in clouds and precipitation: Measurement and modeling challenges 2017; Vol. 58.

[15] Berkemeier T., Shiraiwa M., Pöschl U., Koop T. (2014). Competition between
water uptake and ice nucleation by glassy organic aerosol particles. Atmos. Chem. Phys..

[16] Knopf D. A., Alpert P., Wang B. (2018). The Role of
Organic Aerosol in Atmospheric
Ice Nucleation: A Review. ACS Earth Space Chem..

[17] Wilson T. W., Murray B., Wagner R., Möhler O., Saathoff H., Schnaiter M., Skrotzki J., Price H., Malkin T., Dobbie S., Al-Jumur S. (2012). Glassy aerosols with
a range of compositions nucleate ice heterogeneously at cirrus temperatures. Atmos. Chem. Phys..

[18] Shiraiwa M., Li Y., Tsimpidi A. P., Karydis V. A., Berkemeier T., Pandis S. N., Lelieveld J., Koop T., Poeschl U. (2017). Global distribution
of particle phase state in atmospheric secondary organic aerosols. Nat. Commun..

[19] Gervasi N. R., Topping D. O., Zuend A. (2020). A predictive group-contribution model
for the viscosity of aqueous organic aerosol. Atmos. Chem. Phys..

[20] Maclean A. M., Li Y., Crescenzo G. V., Smith N. R., Karydis V. A., Tsimpidi A. P., Butenhoff C. L., Faiola C. L., Lelieveld J., Nizkorodov S. A., Shiraiwa M., Bertram A. K. (2021). Global distribution
of the phase state and mixing times within secondary organic aerosol
particles in the troposphere based on room-temperature viscosity measurements. ACS Earth Space Chem..

[21] Li Y., Carlton A. G., Shiraiwa M. (2021). Diurnal and
Seasonal Variations in
the Phase State of Secondary Organic Aerosol Material over the Contiguous
US Simulated in CMAQ. ACS Earth Space Chem..

[22] Zhang Z., Li Y., Ran H., An J., Qu Y., Zhou W., Xu W., Hu W., Xie H., Wang Z., Sun Y., Shiraiwa M. (2024). Simulated phase state and viscosity of secondary organic
aerosols over China. Atmos. Chem. Phys..

[23] Luu R., Schervish M., June N., O’Donnell S., Jathar S., Pierce J., Shiraiwa M. (2025). Global Simulations
of Phase State and Equilibration Time Scales of Secondary Organic
Aerosols with GEOS-Chem. ACS Earth Space Chem..

[24] Rasool Q. Z., Shrivastava M., Octaviani M., Zhao B., Gaudet B., Liu Y. (2021). Modeling Volatility-Based Aerosol Phase State Predictions in the
Amazon Rainforest. ACS Earth Space Chem..

[25] Zhang Y., Chen Y. Z., Lambe A. T., Olson N. E., Lei Z. Y., Craig R. L., Zhang Z. F., Gold A., Onasch T. B., Jayne J. T., Worsnop D. R., Gaston C. J., Thornton J. A., Vizuete W., Ault A. P., Surratt J. D. (2018). Effect of the Aerosol-Phase
State on Secondary Organic Aerosol Formation from the Reactive Uptake
of Isoprene-Derived Epoxydiols (IEPDX). Environ.
Sci. Technol. Lett..

[26] Schmedding R., Rasool Q. Z., Zhang Y., Pye H. O. T., Zhang H., Chen Y., Surratt J. D., Lopez-Hilfiker F. D., Thornton J. A., Goldstein A. H., Vizuete W. (2020). Predicting secondary
organic aerosol phase state and viscosity and its effect on multiphase
chemistry in a regional-scale air quality model. Atmos. Chem. Phys..

[27] Maclean A. M., Butenhoff C. L., Grayson J. W., Barsanti K., Jimenez J. L., Bertram A. K. (2017). Mixing
times of organic molecules within secondary
organic aerosol particles: a global planetary boundary layer perspective. Atmos. Chem. Phys..

[28] Bey I., Jacob D. J., Yantosca R. M., Logan J. A., Field B. D., Fiore A. M., Li Q. B., Liu H. G. Y., Mickley L. J., Schultz M. G. (2001). Global modeling
of tropospheric chemistry with assimilated
meteorology: model description and evaluation. J. Geophys. Res.-Atmos..

[29] Philip S., Martin R., Keller C. (2016). Sensitivity
of chemistry-transport
model simulations to the duration of chemical and transport operators:
a case study with GEOS-Chem v10–01. Geoscientific
Model Development.

[30] Pye H. O. T., Seinfeld J. H. (2010). A global perspective
on aerosol from low-volatility
organic compounds. Atmos. Chem. Phys..

[31] Marais E. A., Jacob D. J., Jimenez J. L., Campuzano-Jost P., Day D. A., Hu W., Krechmer J., Zhu L., Kim P. S., Miller C. C., Fisher J. A., Travis K., Yu K., Hanisco T. F., Wolfe G. M., Arkinson H. L., Pye H. O. T., Froyd K. D., Liao J., McNeill V. F. (2016). Aqueous-phase mechanism
for secondary organic aerosol formation from isoprene: application
to the southeast United States and co-benefit of SO2 emission controls. Atmos. Chem. Phys..

[32] Bates K. H., Jacob D. J. (2019). A new model mechanism
for atmospheric oxidation of
isoprene: global effects on oxidants, nitrogen oxides, organic products,
and secondary organic aerosol. Atmos. Chem.
Phys..

[33] Pai S. J., Heald C. L., Pierce J. R., Farina S. C., Marais E. A., Jimenez J. L., Campuzano-Jost P., Nault B. A., Middlebrook A. M., Coe H., Shilling J. E., Bahreini R., Dingle J. H., Vu K. (2020). An evaluation
of global organic aerosol schemes using airborne observations. Atmos. Chem. Phys..

[34] Fisher J. A., Jacob D., Travis K., Kim P., Marais E., Miller C., Yu K., Zhu L., Yantosca R., Sulprizio M., Mao J., Wennberg P., Crounse J., Teng A., Nguyen T., St Clair J., Cohen R., Romer P., Nault B., Wooldridge P., Jimenez J., Campuzano-Jost P., Day D., Hu W., Shepson P., Xiong F., Blake D., Goldstein A., Misztal P., Hanisco T., Wolfe G., Ryerson T., Wisthaler A., Mikoviny T. (2016). Organic nitrate chemistry and its
implications for nitrogen budgets in an isoprene- and monoterpene-rich
atmosphere: constraints from aircraft (SEAC4RS) and ground-based (SOAS)
observations in the Southeast US. Atmos. Chem.
Phys..

[35] Bates K. H., Jacob D., Li K., Ivatt P., Evans M., Yan Y., Lin J. (2021). Development and evaluation of a new compact mechanism
for aromatic oxidation in atmospheric models. Atmos. Chem. Phys..

[36] Gantt B., Johnson M. S., Crippa M., Prevot A. S. H., Meskhidze N. (2015). Implementing
marine organic aerosols into the GEOS-Chem model. Geoscientific Model Development.

[37] Fu, T.-M. ; Jacob, D. J. ; Wittrock, F. ; Burrows, J. P. ; Vrekoussis, M. ; Henze, D. K. Global budgets of atmospheric glyoxal and methylglyoxal, and implications for formation of secondary organic aerosols J. Geophys. Res.-Atmos. 2008; Vol. 113 D15 10.1029/2007JD009505.

[38] Chen Q., Heald C., Jimenez J., Canagaratna M., Zhang Q., He L., Huang X., Campuzano-Jost P., Palm B., Poulain L., Kuwata M., Martin S., Abbatt J., Lee A., Liggio J. (2015). Elemental composition
of organic aerosol: The gap between ambient and laboratory measurements. Geophys. Res. Lett..

[39] McDuffie E. E., Smith S. J., O’Rourke P., Tibrewal K., Venkataraman C., Marais E. A., Zheng B., Crippa M., Brauer M., Martin R. V. (2020). A global anthropogenic
emission inventory of atmospheric
pollutants from sector- and fuel-specific sources (1970–2017):
an application of the Community Emissions Data System (CEDS). Earth System Science Data.

[40] van
der Werf G. R., Randerson J. T., Giglio L., van Leeuwen T. T., Chen Y., Rogers B. M., Mu M. Q., van Marle M. J. E., Morton D. C., Collatz G. J., Yokelson R. J., Kasibhatla P. S. (2017). Global
fire emissions estimates during 1997–2016. Earth System Science Data.

[41] Guenther A. B., Jiang X., Heald C. L., Sakulyanontvittaya T., Duhl T., Emmons L. K., Wang X. (2012). The model
of rmissions
of gases and aerosols from Nature version 2.1 (MEGAN2.1): an extended
and updated framework for modeling biogenic emissions. Geoscientific Model Development.

[42] DeRieux W. S., Li Y., Lin P., Laskin J., Laskin A., Bertram A. K., Nizkorodov S. A., Shiraiwa M. (2018). Predicting the glass transition temperature
and viscosity of secondary organic material using molecular composition. Atmos. Chem. Phys..

[43] Li Y., Day D. A., Stark H., Jimenez J. L., Shiraiwa M. (2020). Predictions
of the glass transition temperature and viscosity of organic aerosols
from volatility distributions. Atmos. Chem.
Phys..

[44] Gregson F. K. A., Gerrebos N. G. A., Schervish M., Nikkho S., Schnitzler E. G., Schwartz C., Carlsten C., Abbatt J. P. D., Kamal S., Shiraiwa M., Bertram A. K. (2023). Phase Behavior
and Viscosity in Biomass Burning Organic Aerosol and Climatic Impacts. Environ. Sci. Technol..

[45] Gerrebos N. G. A., Zaks J., Gregson F., Walton-Raaby M., Meeres H., Zigg I., Zandberg W., Bertram A. (2024). High Viscosity
and Two Phases Observed over a Range of Relative Humidities in Biomass
Burning Organic Aerosol from Canadian Wildfires. Environ. Sci. Technol..

[46] Schnitzler E. G., Gerrebos N. G. A., Carter T. S., Huang Y., Heald C. L., Bertram A. K., Abbatt J. P. D. (2022). Rate
of atmospheric brown carbon
whitening governed by environmental conditions. Proc. Natl. Acad. Sci. U.S.A..

[47] Zhang Y., Nichman L., Spencer P., Jung J., Lee A., Heffernan B., Gold A., Zhang Z., Chen Y., Canagaratna M., Jayne J., Worsnop D., Onasch T., Surratt J., Chandler D., Davidovits P., Kolb C. (2019). The Cooling Rate- and Volatility-Dependent Glass-Forming Properties
of Organic Aerosols Measured by Broadband Dielectric Spectroscopy. Environ. Sci. Technol..

[48] Bateman A. P., Gong Z., Harder T., de Sá S., Wang B., Castillo P., China S., Liu Y., O’Brien R., Palm B., Shiu H., Cirino G., Thalman R., Adachi K., Alexander M., Artaxo P., Bertram A., Buseck P., Gilles M., Jimenez J., Laskin A., Manzi A., Sedlacek A., Souza R., Wang J., Zaveri R., Martin S. (2017). Anthropogenic
influences on the physical state of submicron particulate matter over
a tropical forest. Atmos. Chem. Phys..

[49] O’Brien R. E., Neu A., Epstein S., MacMillan A., Wang B., Kelly S., Nizkorodov S., Laskin A., Moffet R., Gilles M. (2014). Physical properties
of ambient and laboratory-generated secondary organic aerosol. Geophys. Res. Lett..

[50] Lei Z., Chen Y., Zhang Y., Cooke M., Ledsky I., Armstrong N., Olson N., Zhang Z., Gold A., Surratt J., Ault A. (2022). Initial pH Governs Secondary Organic
Aerosol Phase State and Morphology after Uptake of Isoprene Epoxydiols
(IEPDX). Environ. Sci. Technol..

[51] Gorkowski K., Donahue N., Sullivan R. (2020). Aerosol Optical Tweezers Constrain
the Morphology Evolution of Liquid-Liquid Phase-Separated Atmospheric
Particles. CHEM.

[52] Ye Y., Sun J., Fan Y., Li Y., Li Q., He C., Ma S., Zhao Z., Xu T. (2025). Key Roles of Bulk Viscosity and Acidity
on Liquid-Liquid Phase Separation of Atmospheric Organic-Inorganic
Mixed Aerosols. J. Phys. Chem. A.

[53] You Y., Smith M., Song M., Martin S., Bertram A. (2014). Liquid-liquid
phase separation in atmospherically relevant particles consisting
of organic species and inorganic salts. Int.
Rev. Phys. Chem..

[54] Gaston C. J., Riedel T., Zhang Z., Gold A., Surratt J., Thornton J. (2014). Reactive Uptake of an Isoprene-Derived
Epoxydiol to
Submicron Aerosol Particles. Environ. Sci. Technol..

[55] Pankow J.
F. (1994). An absorption
model of the gas/aerosol partitioning involved in the formation of
secondary organic aerosol. Atmosphere Environ..

[56] Li Y., Shiraiwa M. (2019). Timescales
of secondary organic aerosols to reach equilibrium
at various temperatures and relative humidities. Atmos. Chem. Phys..

[57] Andrés
Hernández M. D., Hilboll A., Ziereis H., Förster E., Krüger O., Kaiser K., Schneider J., Barnaba F., Vrekoussis M., Schmidt J., Huntrieser H., Blechschmidt A., George M., Nenakhov V., Harlass T., Holanda B., Wolf J., Eirenschmalz L., Krebsbach M., Pöhlker M., Hedegaard A., Mei L., Pfeilsticker K., Liu Y., Koppmann R., Schlager H., Bohn B., Schumann U., Richter A., Schreiner B., Sauer D., Baumann R., Mertens M., Jöckel P., Kilian M., Stratmann G., Pöhlker C., Campanelli M., Pandolfi M., Sicard M., Gómez-Amo J., Pujadas M., Bigge K., Kluge F., Schwarz A., Daskalakis N., Walter D., Zahn A., Pöschl U., Bönisch H., Borrmann S., Platt U., Burrows J. (2022). Overview:
On the transport and transformation of pollutants in the outflow of
major population centres - observational data from the EMeRGe European
intensive operational period in summer 2017. Atmos. Chem. Phys..

[58] Lin C. Y., Chen W. C., Chien Y. Y., Chou C. C. K., Liu C. Y., Ziereis H., Schlager H., Förster E., Obersteiner F., Krüger O. O., Holanda B. A., Pöhlker M. L., Kaiser K., Schneider J., Bohn B., Pfeilsticker K., Weyland B., Andrés Hernández M. D., Burrows J. P. (2023). Effects of transport on a biomass burning plume from
Indochina during EMeRGe-Asia identified by WRF-Chem. Atmos. Chem. Phys..

[59] Wofsy, S. ; Afshar, S. ; Allen, H. ; Apel, E. ; Asher, E. ; Barletta, B. ; Bent, J. ; Bian, H. ; Biggs, B. ; Blake, D. ATom: Merged atmospheric chemistry, trace gases, and aerosols. ORNL Distributed Active Archive Center 2018 10.3334/ORNLDAAC/1581.

[60] Rastigejev Y., Park R., Brenner M., Jacob D. (2010). Resolving intercontinental
pollution plumes in global models of atmospheric transport. J. Geophys. Res.-Atomspheres.

[61] Pajunoja A., Hu W. W., Leong Y. J., Taylor N. F., Miettinen P., Palm B. B., Mikkonen S., Collins D. R., Jimenez J. L., Virtanen A. (2016). Phase state of ambient aerosol linked with water uptake
and chemical aging in the southeastern US. Atmos.
Chem. Phys..

[62] Saukko E., Lambe A., Massoli P., Koop T., Wright J., Croasdale D., Pedernera D., Onasch T., Laaksonen A., Davidovits P., Worsnop D., Virtanen A. (2012). Humidity-dependent
phase state of SOA particles from biogenic and anthropogenic precursors. Atmos. Chem. Phys..

[63] Kim D., Campuzano-Jost P., Guo H., Day D. A., Yang D., Dhaniyala S., Williams L., Croteau P., Jayne J., Worsnop D., Volkamer R., Jimenez J. L. (2025). Development and
characterization of an aircraft inlet system for broader quantitative
particle sampling at higher altitudes: aerodynamic lenses, beam and
vaporizer diagnostics, and pressure-controlled inlets. Aerosol Res..

[64] Guo H., Campuzano-Jost P., Nault B., Day D., Schroder J., Kim D., Dibb J., Dollner M., Weinzierl B., Jimenez J. (2021). The importance of size ranges in aerosol instrument
intercomparisons: a case study for the Atmospheric Tomography Mission. Atmos. Meas. Tech..

[65] Brock C. A., Froyd K., Dollner M., Williamson C., Schill G., Murphy D., Wagner N., Kupc A., Jimenez J., Campuzano-Jost P., Nault B., Schroder J., Day D., Price D., Weinzierl B., Schwarz J., Katich J., Wang S., Zeng L., Weber R., Dibb J., Scheuer E., Diskin G., DiGangi J., Bui T., Dean-Day J., Thompson C., Peischl J., Ryerson T., Bourgeois I., Daube B., Commane R., Wofsy S. (2021). Ambient aerosol
properties in the remote atmosphere from global-scale in situ measurements. Atmospheric Chem. Phys..

[66] Brock C. A., Williamson C., Kupc A., Froyd K., Erdesz F., Wagner N., Richardson M., Schwarz J., Gao R., Katich J., Campuzano-Jost P., Nault B., Schroder J., Jimenez J., Weinzierl B., Dollner M., Bui T., Murphy D. (2019). Aerosol size
distributions during the Atmospheric Tomography
Mission (ATom): methods, uncertainties, and data products. Atmos. Meas. Tech..

[67] Mu Q., Shiraiwa M., Octaviani M., Ma N., Ding A., Su H., Lammel G., Pöschl U., Cheng Y. (2018). Temperature effect
on phase state and reactivity controls atmospheric multiphase chemistry
and transport of PAHs. Sci. Adv..

[68] Shrivastava M., Lou S., Zelenyuk A., Easter R., Corley R., Thrall B., Rasch P., Fast J., Simonich S., Shen H., Tao S. (2017). Global long-range
transport and lung cancer risk from polycyclic
aromatic hydrocarbons shielded by coatings of organic aerosol. Proc. Natl. Acad. Sci. U.S.A..

[69] Serrano
Damha C., Gorkowski K., Zuend A. (2025). Implications of reduced-complexity
aerosol thermodynamics on organic aerosol mass concentration and composition
over North America. Atmospheric Chem. Phys..

[70] Song M., Jeong R., Kim D., Qiu Y., Meng X., Wu Z., Zuend A., Ha Y., Kim C., Kim H., Gaikwad S., Jang K., Lee J., Ahn J. (2022). Comparison
of phase states of PM2.5 over megacities, Seoul and Beijing, and their
implications on particle size distribution. Environ. Sci. Technol..

