# Supplementary material for: Global Impacts of Simulating Organic Aerosol Phase State
Source: Environ Sci Technol. 2026 Jul 21;60(30):21071–81. doi: 10.1021/acs.est.5c13238 (PMC13449889; doi:10.1021/acs.est.5c13238)
Supplement: Supplementary file 1 [file es5c13238_si_001.pdf]

Supporting Information

(5 text, 13 figures)

Global impacts of simulating organic aerosol phase state

Yumin Li[1,], Colette L. Heald[1,*] Ruqian Miao[2], Pedro Campuzano-Jost[3], Jose L Jimenez[3]*

[1]Institute for Atmospheric and Climate Science, ETH Zurich, 8092 Zurich, Switzerland

[2]State Key Laboratory of Regional Environment and Sustainability, College of Environmental Sciences and Engineering, Peking University, Beijing, 100871, China

[3]Cooperative Institute for Research in Environmental Sciences and Department of Chemistry, University of Colorado, Boulder, CO, 80309, USA

* Corresponding authors: yumin.li@env.ethz.ch and colette.heald@env.ethz.ch

List of Texts, Tables, and Figures

Text S1. Details of the GEOS-Chem model, organic aerosol simulation and the emission inventories used in this study.

Text S2. Uncertainty in glass transition temperature and viscosity

Text S3. Uncertainty in phase state dependent OA reaction

Text S4. Uncertainty in OA phase state impact

Text S5. Uncertainty associate with aircraft AMS PM_1 inlet

Figure S1. Predicting viscosity change with T_g under different ambient temperatures.

Figure S2. Simulated global annual (2017) mean high altitude OA viscosity. (A) at 7 km (B) at 2.5 km.

Figure S3. Simulated global annual (2017) mean RH and temperature at different altitudes. (A1-A3) RH at 7 km, 2.5 km and surface,(B1-B3) temperature at 7 km, 2.5 km and surface.

Figure S4. Simulated global annual (2017) mean source specific OA concentration at different attitudes. (A1-A3) total OA; (B1-B3) hydrophobic POA; (C1-C3) hydrophilic POA; (D1-D3) biogenic SOA; (F1-F3) anthropogenic SOA.

Figure S5. Sensitivity simulation mean source specific OA and total OA concentration change due to phase state effects at 3 specific altitudes under (A) high-end viscosity assumptions and (B) low-end viscosity assumptions.

Figure S6. Comparison of simulated mean total OA vertical concentration profiles with and without phase state treatments against aircraft measurement data along with the aircraft maps. (A) East Asia, (B) Remote ocean.

Figure S7. Comparison of simulated mean OA vertical concentration profiles with and without phase state treatments against aircraft measurement data. Simulated OA concentration profiles with (A1) and without (A2) phase state *vs* measurement from ATom1 to ATom4 campaign in remote ocean areas. The Pearson correlation coefficients (r) and normalized mean bias (NMB) are displayed in the inset. Obs_AMS indicate the AMS measured concentrations (black line), Obs_AMS+PALMS indicate the devided $PM_{2.5}$ OA concentrations from AMS and PALMS measurement (grey line) [1], and the Obs_SD indicate the standard deviation of the observations in each altitude bin. The aircraft flight tracks are also shown in the inset.

Figure S8. redicted dry SOA T_g under different molecular weight and O:C ratio. The rang of molecular weight and O:C ratio get from Chen, et al. [2], and the T_g is calculated following Shiraiwa, et al. [3].

Figure S9. Sensitivity of predicted $T_{g,total}$ to external mixing calculations using dry versus hygroscopic-growth-adjusted OA mass weighting in Eq. 2.

Figure S10. Simulated meridional mean source specific OA and total OA T_g. (A) hydrophilic POA, (B) hydrophobic POA, (C) biogenic SOA, (D) anthropogenic SOA, (E) total OA.

Figure S11. Predicted γ under different particle radius (r) and morphology assumptions, including homogeneous particles and core-shell particles with shell thicknesses ranging from 0.1 to 0.9 times of the particle radius. (A1) γ for isoprene SOA, r = 100 nm ; (A1) γ for isoprene SOA, r = 10 nm ; (A1) γ for isoprene SOA, r = 1000 nm.

Figure S12. (A) Temperature-dependent C* and predicted viscosity across model grid boxes at different altitudes. (B) Simulated OA mass fraction under different temperature-dependent C* and viscosity conditions, along with the controlling scheme (New phase-state scheme or VBS scheme). Unshaded regions indicate OA ranges that can be represented by the corresponding schemes, while

gray-shaded regions denote conditions that cannot be accurately captured for condensation or evaporation scenarios. 2% and 17% indicate fraction of simulated OA mass fraction in the respective gray-shaded regions.

Figure S13. Predicted τ_{mix} change with viscosity under different particle radius asumptions (*r* =100 nm; *r*=10 nm; *r* =1000 nm).

References (*1-63*)

S1. Details of the GEOS-Chem model, organic aerosol simulation, and the emission inventories used in this study

S1.1 GEOS-Chem and Organic aerosol simulation

GEOS-Chem included a comprehensive HO_x-NO_x-VOC-ozone-halogen-aerosol mechanism for tropospheric chemistry.[4-6] Aerosols in the standard GEOS-Chem model included dust, sea salt, elemental carbon aerosol, primary OC, secondary inorganic aerosol (sulfate, nitrate, ammonium), and secondary organic aerosol.[7-10]

We use the complex OA schemes that incorporate the semi-volatile primary organic aerosol (POA) simulation in GEOS-Chem[10-15]. All freshly emitted POA from anthropogenic and biomass burning are emitted as hydrophobic gas phase (EPOG) and allowed to reversibly partition to the hydrophobic aerosol phase (EPOA). The EPOG undergoes OH oxidation in the gas phase, producing oxidized hydrophilic organic gas (OPOG), which can also reversibly partition into oxidized hydrophilic aerosol phase (OPOA).

We employ the Volatility Basis Set scheme [11] to form SOA from anthropogenic precursors and terpenes. Irreversible uptake schemes are used for the formation of isoprene SOA[12], organic nitrate SOA from both anthropogenic and biogenic precursors[14, 15] and glyoxal and methylglyoxal SOA[16]. The differences in treatment reflect these processes were implemented in GEOS-Chem following distinct assumptions from the respective reference studies.

The transport of atmospheric constituents in GEOS-Chem was simulated using the TPCORE advection algorithm.[17] Dry deposition of aerosols and gases was simulated by the resistance-in-series scheme.[18, 19] Wet deposition of aerosols and gases was calculated as described in Liu, et al. [20] and Amos, et al. [21]. The scavenging efficiency for fine, hydrophilic particulates was assumed to be unity.

S1.2 Emission

Global monthly anthropogenic emissions of primary organic carbon (OC) and other precursors follow the Community Emissions Data System (CEDS, native resolution 0.3125° longitude × 0.25° latitude, https://esgf-node.llnl.gov/search/input4mips/) [22], but are superseded by regional inventories: the National Emissions Inventory 2016 for the U.S. (NEI16, https://www.epa.gov/air-emissions-inventories/national-emissions-inventory-nei), the Air Pollutants Emissions Inventory for Canada (APEI, http://ec.gc.ca/inrp-npri/donnees-data/ap), the Diffuse and Inefficient Combustion Emissions inventory for Africa (DICE-Africa)[23], and the Multi-resolution Emission Inventory for China (MEIC) version 1.4 [24]. Global monthly year-specific biomass burning emissions, including small fires, are taken from the Global Fire Emissions Database (GFEDv4.1s, native resolution 0.25°, http://www.globalfiredata.org) [25]. Biogenic VOC emissions are using the offline MEGAN (v2.1) algorithm [26]. We use the year 2017 emissions for the standard global simulation, and year-specific emissions are employed when comparing with aircraft observations.

S2 Uncertainty in glass transition temperature and viscosity

S2.1 Glass transition temperature (T_g)

We utilize the glass transition temperature (T_g) to calculate OA viscosity (Eq. 1). T_g represents the temperature at which the viscosity of OA reaches 10^{12} Pa·s, marking the transition of OA to a solid phase.

There are few experimental studies constraining the phase state of POA. Only two studies have reported direct laboratory measurements of both T_g and their variation with RH for hydrophilic and hydrophobic bulk biomass burning OA[27, 28]. These laboratory measurements focus predominantly on fresh POA. However, laboratory studies examining specific wood types (e.g., pine, poplar) consistently show that aged OA samples exhibit 2.5-13 times higher viscosity than fresh OA samples under identical conditions [29]. The sole field measurement of biomass burning OA also supports this finding, though methodological limitations yielded only an approximate viscosity range rather than precise values [30]. Moreover, dilution of smoke plumes preferentially removes low-molecular-weight, low-polarity compounds, enriching the residual aerosol in larger, more polar species with higher viscosity, thereby increasing the bulk viscosity of POA[31].

For anthropogenic OA, the chemical composition is complex due to multiple emission sources. POA emitted from residential combustion, such as wood burning, may exhibit properties similar to biomass burning OA. Hydrocarbon-like organic aerosol (HOA) from traffic emissions generally has a significantly lower viscosity than biomass burning OA [32].

For SOA, three different methods exist to predict dry T_g: using molecular weight and the O:C ratio [3, 33], using the O:C ratio and saturation mass concentration [34], and based on the O:C ratio and vapor pressure [35]. All of these methods results are consistent[3, 33-35]. We choose to use molecular weight and the O:C ratio to predict T_g and this method predicts higher viscosity for species with

larger molecular weights and O:C ratios. We estimated dry SOA T_g by using a molecular weight of 250 g mol^{-1}, which is a representative value within the commonly assumed range of 150-300 g mol^{-1} in atmospheric modeling. We also used O:C ratios of 0.7 and 1.1 for biogenic and anthropogenic SOA, respectively. These values are consistent with the typical ranges observed in the literature for biogenic (0.2-1.1) and anthropogenic (0.6-1.5) SOA[2]. This approach introduces uncertainty in the predicted T_g values. To address this, a sensitivity simulation spanning molecular weights from 100 to 300 g mol^{-1} and O:C ratios from 0.2 to 1.8 predicts dry T_g values ranging from 130 to 390 K (Fig. S8). Our chosen values lie near the middle of this possible range.

During the aging of SOA, both the molecular weight and the O:C ratio tends to increase, which can lead to higher viscosity. However, we do not account for this evolution in our simulations, but our selected O:C values represent values across fresh and aged samples.

S2.2 Environmental effect on T_g

The T_g of OA is significantly influenced by relative humidity (RH) [36, 37]. Water can act as a plasticizer, lowering the overall T_g when mixed with OA. In our standard simulation, we utilize the laboratory-measured RH-dependent T_g for POA [27]. Experimental measurements indicate that the T_g of hydrophilic POA decreases as RH increases from 0% to 100%, while the T_g of hydrophobic OA exhibits minimal change until RH exceeds 95% [27].

For secondary OA we use the Gordon-Taylor-approach [36]: Eq. S1, to calculate T_g after mixing with water.

$$T_g = \frac{w_{org}\, T_{g,org} \frac{1}{k_{GT}} + (1 - w_{org})\, T_{g,water}}{(1 - w_{org}) + \frac{1}{k_{GT}} w_{org}} \qquad \text{S1}$$

Where the $T_{g,org}$ and $T_{g,water}$ = 136 K are the T_g for dry OA species and for water respectively, k_{GT} is the Gordon–Taylor parameter which assume as 2.5 ± 1 is an empirical parameter derived from extensive experimental fitting [3, 36, 38]. $w_{org} = \frac{m_{SOA}}{m_{SOA}+m_{water}}$ is the organic mass fraction of SOA where m_{SOA} and m_{water} are the mass concentration of SOA and water respectively. m_{water} can be calculated based on RH and m_{SOA} [39]: Eq. S2.

$$m_{water} = \frac{\kappa \rho_{water} m_{SOA}}{\rho_{SOA}(\frac{1}{a_w}-1)} \qquad \text{S2}$$

Where the density of water (ρ_w) and SOA particles (ρ_{SOA}) are assumed to be 1.0 g cm^{-3} and 1.4 g cm^{-3} (1.2 to 1.5), respectively [40-42]. a_w is the water activity calculated as RH/100. The effective hygroscopicity parameter (κ) for SOA is assumed to be 0.1 based on measurements[43-45] and consistent with previous simulations and similar with GEOS-Chem default values[3, 46, 47].

Inorganic aerosols, such as sulfates and nitrates also can act as plasticizers [37, 48] to lower the overall T_g when mixed with OA. However, observations indicate that solid, semi-solid OA and inorganic aerosols typically in phase seperate, although its occurrence can depend on RH and composition [37, 49-52]. Therefore, we assume to consider that OA and inorganic aerosols exist externally mixing in separate phases and ignore the impact of inorganic aerosols on T_g on our standard simulation. This assumption may lead to overestimation of OA viscosity typical in the high RH regime[48]. Previous studies have also shown that considering internal mixing between SOA and inorganic species can increase the frequency of liquid-state OA by 5-45% in China, with the largest effects occurring in regions characterized by high RH and low OA viscosity [47].

We assume external mixing between source-specific OA when calculating the T_g of total OA, as the detailed mixing state remains highly uncertain and internal mixing would introduce additional unconstrained variables. In Eq. 2, we use dry masses of source-specific OA to compute the mass-weighted mean T_g of total OA. If the water mass associated with each OA source is

considered by using hygroscopic growth factors in standard GEOS-Chem, the relative mass contributions of different OA components would change slightly. However, this would only lead to a minor change (<1 K) in the resulting $T_{g,total}$ (Fig. S9). We also note that, these assumptions are independent of how species are tracked within the model framework. Additionally, in our phase state dependent OA simulation, chemical reactions are parameterized using source-specific OA viscosities for each OA component. Thus, the mass-weighted total OA viscosity is not used in any further calculations and does not influence reaction rates.

S2.3 Sensitivity between viscosity and T_g

Based on the above assumptions, our final predicted T_g range from 148K to 326 K (Fig. S10). We conducted a sensitivity simulation to investigate the impact of T_g variation on the predicted viscosity in this range (Fig. S1). The viscosity prediction exhibits notable sensitivity to T_g in the semi-solid state. When T_g is low and the OA in the liquid state or a low-viscosity semi-solid state , a 50 K change in T_g results in only a 1-2 order of magnitude changes in viscosity. Conversely, when T_g is elevated and the OA presents a high-viscosity semi-solid state, a 50 K variation in T_g can induce a 6-8 order of magnitude change in viscosity. However, for OA in the solid state, viscosity changes negligibly influence subsequent chemical processes, as chemical reactions are nearly quiescent under such conditions.

S3 Uncertainty in phase state dependent OA reaction

S3.1 Sensitivity simulations of γ for different morphologies

Given that particles exhibit complex morphologies, there are two typical approaches to assessing the impact of viscosity on the reactive uptake process: (1) a well-mixed homogeneous OA, as described in Eq. S3 [53, 54]; (2) a core-shell structure with a liquid phase core and a high-viscosity OA coating, as described in Eq. 4 [55, 56]. We adopt the core-shell assumption to calculate the γ in the standard simulation, as prior studies have established that OA tends to exist in a phase separated state which forms a highly viscous shell with a liquid core [36, 49, 52]. To quantify the sensitivity of γ to viscosity under these assumptions, we conducted systematic simulations with shell thicknesses ranging from 0.1 to 0.9 times the particle radius for the core-shell structures and for the homogeneous structure for isoprene SOA (Fig. S11). Our findings reveal that under a thin-shell core-shell structure (1/10 radius), the variation of γ with viscosity closely approximates that of the homogeneous structure, whereas thicker shells induce a more significant decrease in γ when viscosity increases diffusion-limited transport through the viscous phase (Fig. S11). Ultimately, we adopted the core-shell structure assumption with a thin shell (1/10 radius) to calculate the viscosity-dependent γ in our simulations as it closely aligns with physical realism[56] and yields results similar to those obtained with the homogeneous structure assumption.

Our sensitivity simulations further demonstrate that a larger particle radius (*r*), induces more pronounced reductions of γ (Fig. S11). Choosing r = 100 nm in our standard simulation following previous studies to calculate γ is a reasonable approach, as it allows γ to start decreasing when viscosity approaches near 10^2 Pa·s, corresponding to the onset of semisolid phase states. While a larger *r* would exaggerate the sensitivity of γ to viscosity changes, smaller *r* would weaken the effect of viscosity on γ. Furthermore, near the boundary between the liquid state and the semi-

liquid state, sensitivity to changes in *r* is greater than that in the high viscosity semi-solid and solid states.

$$\frac{1}{\gamma} = \frac{1}{\alpha} + \frac{r\omega}{4D_g} + \frac{\omega}{4RTH^*_{org}\sqrt{D_{org}k_{aq}}}\left(\frac{1}{coth(q_{aq}) - \frac{1}{q_{aq}}}\right) \quad \text{S3}$$

Where α is the mass accommodation coefficient (taken as 1), D_g is the gas-phase diffusion coefficient (0.1 $cm^2\ s^{-1}$), ω is mean gas-phase molecular speed (cm s^{-1}), *r* is the aerosol particle radius (cm), R is the universal gas constant (0.08206 L atm $K^{-1}\ mol^{-1}$), T is temperature (K), H^*_{org} is effective Henry's Law constant (M atm^{-1}), k_{aq} is pseudo-first-order aqueous-phase reaction rate constant (s^{-1}), $q_{aq} = R_p\sqrt{k_{aq}/D_{org}}$. D_{org} is the diffusion constant ($m^2\ s^{-1}$) is calculated from Eq. 3 as viscosity dependent.

S3.2 Sensitivity simulations of gas-particle partitioning

Our phase-state-dependent gas-particle partitioning scheme is built on the standard GEOS-Chem VBS framework. We isolate the effect of phase state by comparing simulations with and without the phase state dependent treatment. We do not change the equilibrium state from the VBS scheme but add an additional phase state dependent delay timescale (τ_p) when the particle mixing timescale exceeds one chemistry time step. In the following subsections, we discuss in detail the potential uncertainties associated with the underlying VBS scheme (Text S3.2.1), volatility dependent equilibration behavior (Text S3.2.2), and OA mass related effects (Text S3.2.3).

<u>S3.2.1 Uncertainty associated with VBS scheme</u>

Firstly, the standard VBS scheme, which is widely used in three-dimensional models such as GEOS-Chem, WRF-Chem, CESM, and CMAQ, assumes that gas-particle partitioning reaches

equilibrium within one chemistry timestep, without explicitly calculating the equilibration timescale. The equilibrium partitioning of organic species is diagnosed from the temperature-dependent volatility (C*) and the available absorbing organic mass in each chemistry time step (20 min). This assumption is generally appropriate for organics with high volatility and low viscosity, which account for more than half of the OA mass in our simulations. Accordingly, the standard VBS scheme continues to be applied to low viscosity organics in our simulations (Fig. S12).

However, based on estimated condensation and evaporation timescales from Li and Shiraiwa [56]. The VBS scheme tends to underestimate the equilibration time of low volatility organics during evaporation process. This limitation is related to the volatility dependence of equilibration rather than particle phase state itself. Therefore, this bias does not alter our evaluation of phase state impacts based on the comparison between simulations with and without the phase-state treatment, although it remains a limitation of the underlying VBS framework. The VBS scheme tends to underestimate the equilibration time for all high viscosity organics.

In addition, uncertainties in the VBS scheme are closely tied to how volatility is represented. The partitioning state is determined by temperature dependent C* and OA mass. The temperature dependence of C* is calculated from the assumed reference C* bins at 298 K and the enthalpy (ΔH). In standard GEOS-Chem, the assumed reference C* values at 298 K range from 0.1 to 1000 $\mu g\ m^{-3}$, while species with reference C* below 0.1 $\mu g\ m^{-3}$ are assumed to remain entirely in the particle phase. GEOS-Chem further assumes a ΔH value of 42 $kJ\ mol^{-1}$, which is in the middle of the typical range for SOA species. Overall, for under low viscosity conditions, the uncertainty introduced by the VBS scheme is mainly associated with low volatility OA; species with lower reference C* values and larger ΔH would likely involve greater uncertainty.

S3.2.2 Simulated OA mass across volatility and viscosity controlled regimes

Gas-particle equilibration is influenced not only by particle viscosity, but also by volatility. Therefore, the phase state dependent gas-particle partitioning scheme, which constrains the timescale of approach to equilibrium using viscosity, is expected to provide reliable predictions of OA partitioning only when phase state is the dominant kinetic limitation.

Figure S12 summarizes the model distribution of temperature-dependent C* and predicted viscosity across grid boxes at different altitudes (panel A), together with the simulated OA mass fraction associated with each regime (panel B). Panel B can be compared to timescales for condensation and evaporation scenarios with Li and Shiraiwa [56], because the relative importance of viscosity and volatility differs between these two processes.

If only the standard VBS scheme is applied, all species are treated as liquid, and the equilibration timescale of highly viscous OA is therefore underestimated. Introducing our phase-state-dependent scheme improves the representation of this delay for highly viscous OA, but two regimes (gray-shaded in Fig. S12B) remain that are not fully captured by the model treatment.

For evaporation, the standard VBS framework cannot capture the long evaporation timescales that may occur for low volatility species even under low viscosity conditions. In this regime, the timescale is controlled mainly by volatility rather than particle-phase diffusion, and neglecting this effect may lead to an underestimation of OA mass. This limitation affects both our standard and phase-dependent schemes, and thus the relative viscosity effect estimated in our study should be unaffected for these low-viscosity, low-volatility grid boxes, which account for 17% of the simulated OA mass. However, our phase-state-dependent scheme likely corrects this bias for the high-viscosity, low-volatility population, and this would overestimate the impact of phase state itself. These grid boxes account for about 3% of the simulated OA mass.

For condensation, our phase-state-dependent scheme may overestimate equilibration timescales under conditions of high viscosity and low volatility. This would tend to bias OA concentrations low. However, these grid boxes account for only about 2% of the simulated OA mass fraction and therefore do not appreciably affect our results. Instead, they suggest that the simulated OA concentrations should be interpreted as conservative estimates. This is particularly relevant in the upper troposphere, where actual OA concentrations may be higher than our simulation indicates.

Further improvement in gas-particle partitioning would require a framework that explicitly distinguishes condensation from evaporation and resolves their different kinetic controls. However, it is currently not feasible to represent such processes explicitly in the global model framework. In addition, a more explicit partitioning parameterization would require better constraints on gas and particle chemistry, as well as the associated physicochemical properties, before it can be accurately implemented in global three-dimensional models[63].

S3.2.3 Regimes with uncertainty associated with OA mass loading

OA mass loading can also affect the equilibration time and thus represents an additional source of uncertainty beyond viscosity and volatility.

In the low visocisity regime in which the standard VBS scheme is applied, the VBS scheme can capture the equilibration timescale for all high volatility organics across the relevant OA mass range. For low volatility organics, the VBS scheme also captures the equilibration timescale over most of the atmospheric relevant range, except under extremely low concentration conditions [58, 62].

The high viscosity regime in which the phase state treatment is applied generally occurs under relatively low absorbing organic mass conditions (typically < 1 μg m^{-3}; Fig. 1, Figs. S2 and S4). In this regime, the equilibration time of high volatility organics is not sensitive to organic mass,

and the equilibration time of low volatility organics varying within several-fold time[58, 62] across the relevant mass range. Overall, the variation associated with organic mass is smaller than the effects of viscosity and volatility.

S3.2.4 Sensitivity simulation for τ_{mix} with different *r* values

We also conduct a sensitivity simulation for τ_{mix} when considering different *r* assumptions (Fig. S13). Our result indicates that assuming a larger *r* will lead to a longer τ_{mix} at the same viscosity condition, which is consistent with previous study[36]. A one order of magnitude increase in *r* results in a two order of magnitude increase in τ_{mix}.

S4 Uncertainty in OA phase state impact

Considering that the predicted POA and SOA T_g (Text S2) and the corresponding OA viscosity predicted as a function of T_g under ambient temperatures are subject to uncertainties (Fig. S1), these uncertainties ultimately affect the assessment of phase state impacts on OA concentrations. To address this, we designed two sensitivity simulations using high-end and low-end viscosity assumptions.

In the high-end assumption, we applied the upper-bound dry T_g values for both hydrophilic POA and SOA (hydrophilic POA dry T_g = 290 K; biogenic SOA T_g = 263.46 K; anthropogenic SOA T_g = 297.91 K), whereas in the low-end assumption, we used the lower-bound T_g values for hydrophilic POA and SOA (hydrophilic POA T_g = 240.51 K; biogenic SOA T_g = 339.25 K; anthropogenic SOA T_g = 373.7 K). For hydrophobic POA, only one reported value is currently available (Text S2). Given that hydrophobic POA contributes much less to the total OA concentration than hydrophilic OA, we did not change the hydrophobic POA T_g in our sensitivity simulation.

The predicted total OA viscosity under the high-end and low-end assumptions is shown in Fig.2B, 2C as orange uncertainty bars. Although both sensitivity simulations exhibit strong correlations with the observations, comparable to the standard case (r = 0.9), the high-end assumption clearly overestimates the observed viscosity (NMB = 53%), whereas the low-end assumption underestimates the observation (NMB = -58%).

Figure S5 illustrates the global mean vertical OA concentration responses of POA and SOA to phase state in our sensitivity simulations. Under the high-end viscosity assumption, the phase state

impact is more pronounced than our standard simulation, with 38% decrease in surface concentration and a 3% decrease at 7km, resulting in an over all 35% reduction in the vetical concentration gradient. Under the low-end viscosity assumption, the phase state impact is weaker than our standard simulation, with a 10% decrease in surface concentration and 4% increase at 7km, leading to an over all 14% reduction in the vertical concentration gradient. Our low-end sensitivity simulation may substantially underestimate the phase state impact on gas-particle partitioning (especially POA), given that the GEOS-Chem model time step is 20 min and lower viscosities may not effectively limit partitioning within this timescale and thus may be numerically neglected.

Overall, in all cases, accounting for phase state consistently reduces the vertical gradient in OA concentrations, weakening the decrease in concentration from the surface to higher altitudes. These findings are consistent with our main conclusions.

S5 Uncertainty associate with aircraft AMS PM_1 inlet

AMS measurement with a PM_1 inlet as used in EMERGE-AS and ATom campaigns, may systematically underestimate fine ($PM_{2.5}$) OA concentration[59, 60]. For the EMERGE-AS campaign, $PM_{2.5}$ OA data are not yet available for comparison. For ATom, previous studies suggest that AMS measurements may underestimate fine OA by up to 30% based on instrument comparisons[1, 61]. In addition, for ATom, $PM_{2.5}$ OA concentrations can be indirectly derived from PLAMS and AMS data (Fig. S7)[1]. However, these indirect $PM_{2.5}$ OA estimates may be subject to potential positive bias, because negative values were set to zero during the calculation. In our simulations, incorporating OA phase state effects also improves the agreement with the derived $PM_{2.5}$ OA concentrations, with the increasing from $r = 0.56$ to $r = 0.80$. Our simulated OA concentrations lie between the AMS measurements and the AMS+PLAMS-derived concentrations, providing further confidence in the robustness of our approach.

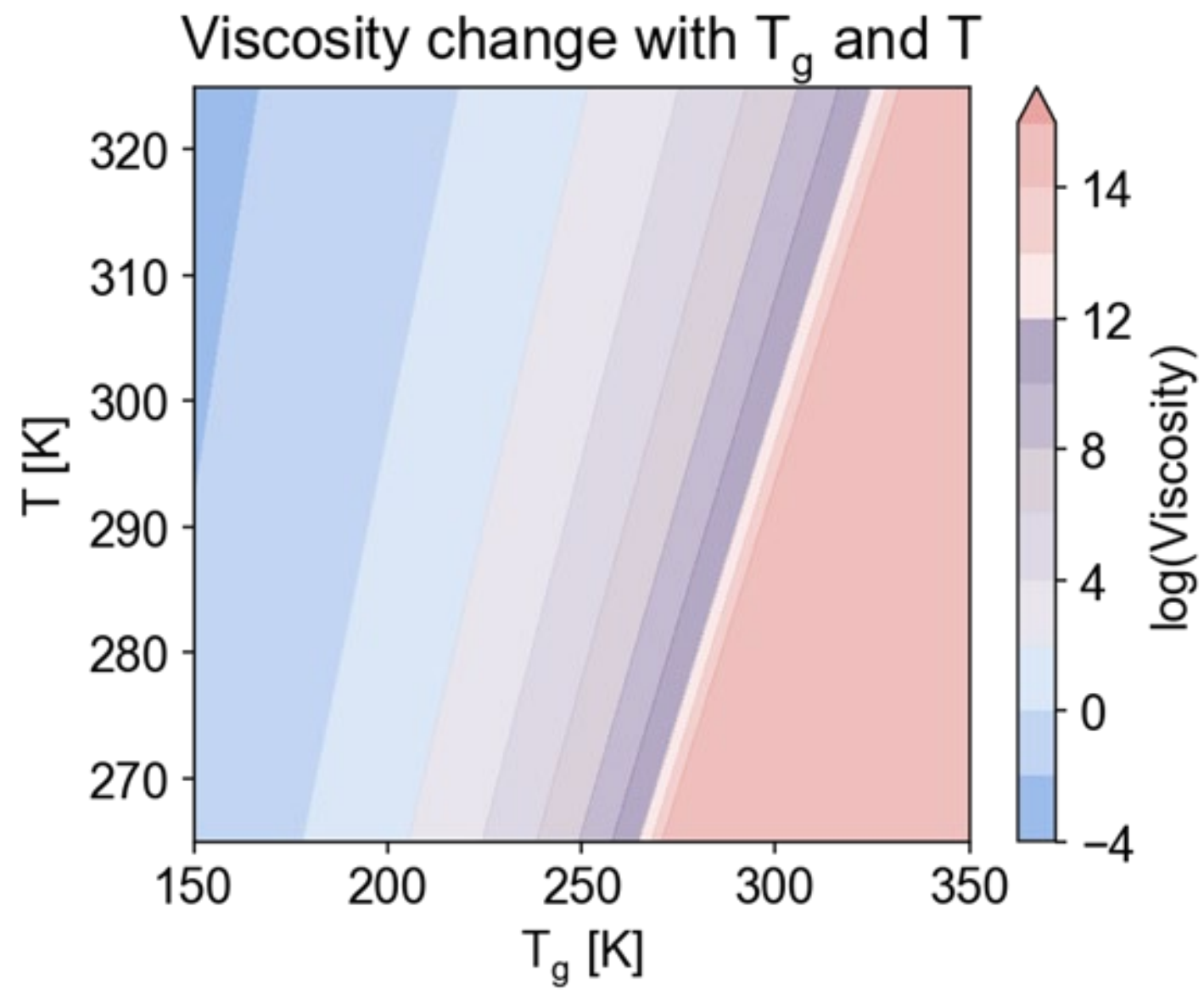

Figure S1. Predicting viscosity change with T_g under different ambient temperatures.

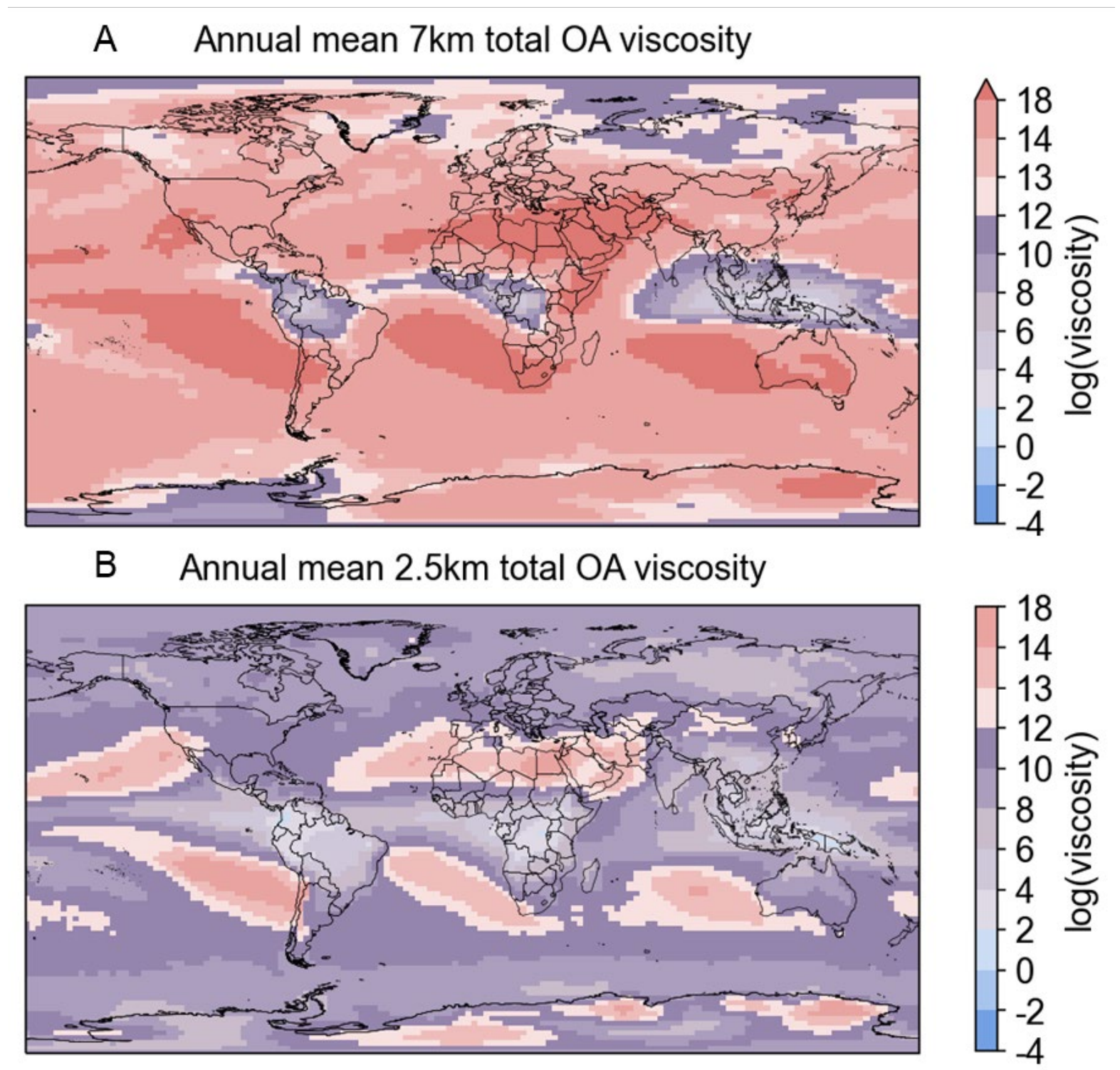

Figure S2. Simulated global annual (2017) mean high altitude OA viscosity. (A) at 7 km (B) at 2.5 km.

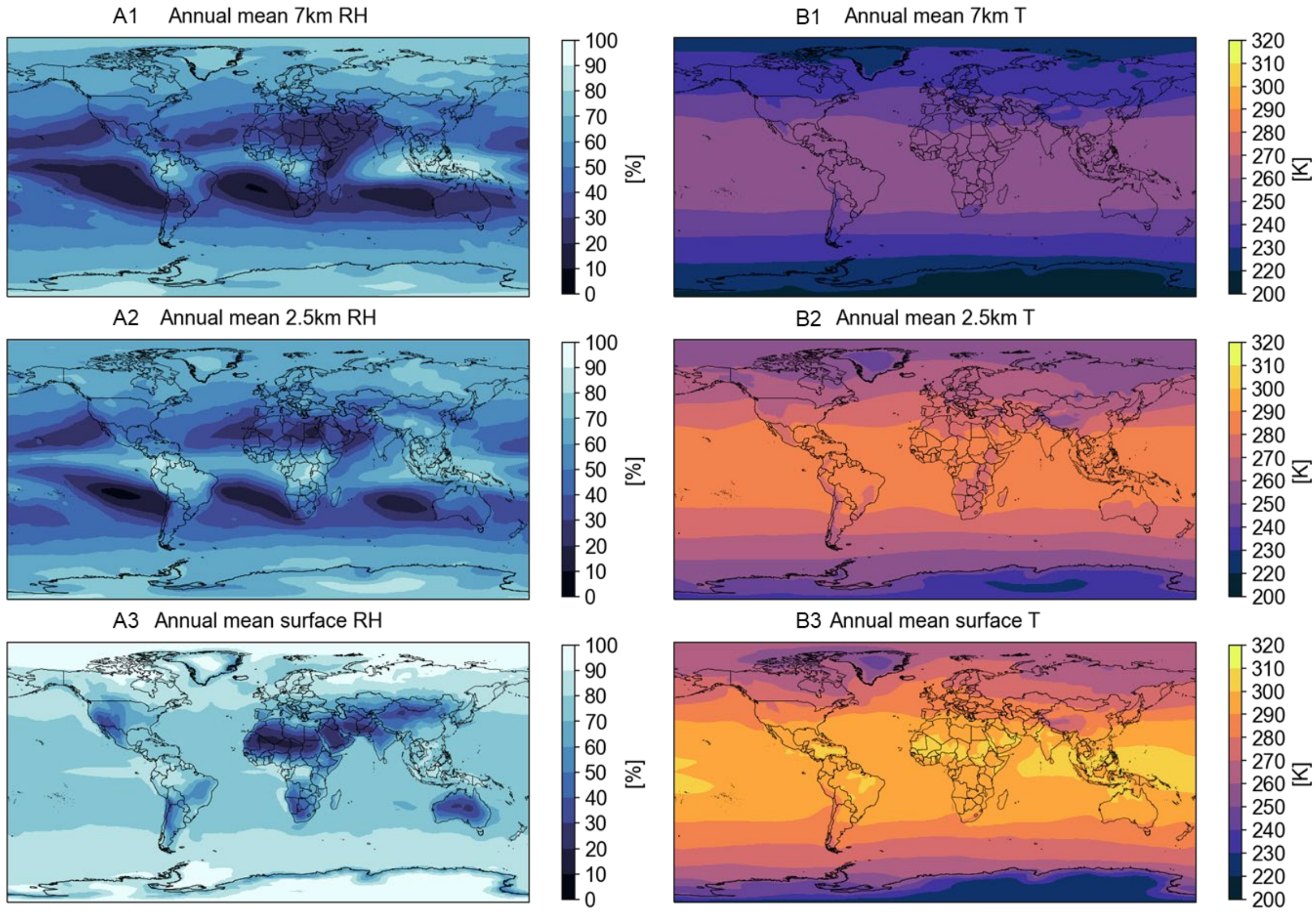

Figure S3. Simulated global annual (2017) mean RH and temperature at different altitudes. (A1-A3) RH at 7 km, 2.5 km and surface,(B1-B3) temperature at 7 km, 2.5 km and surface.

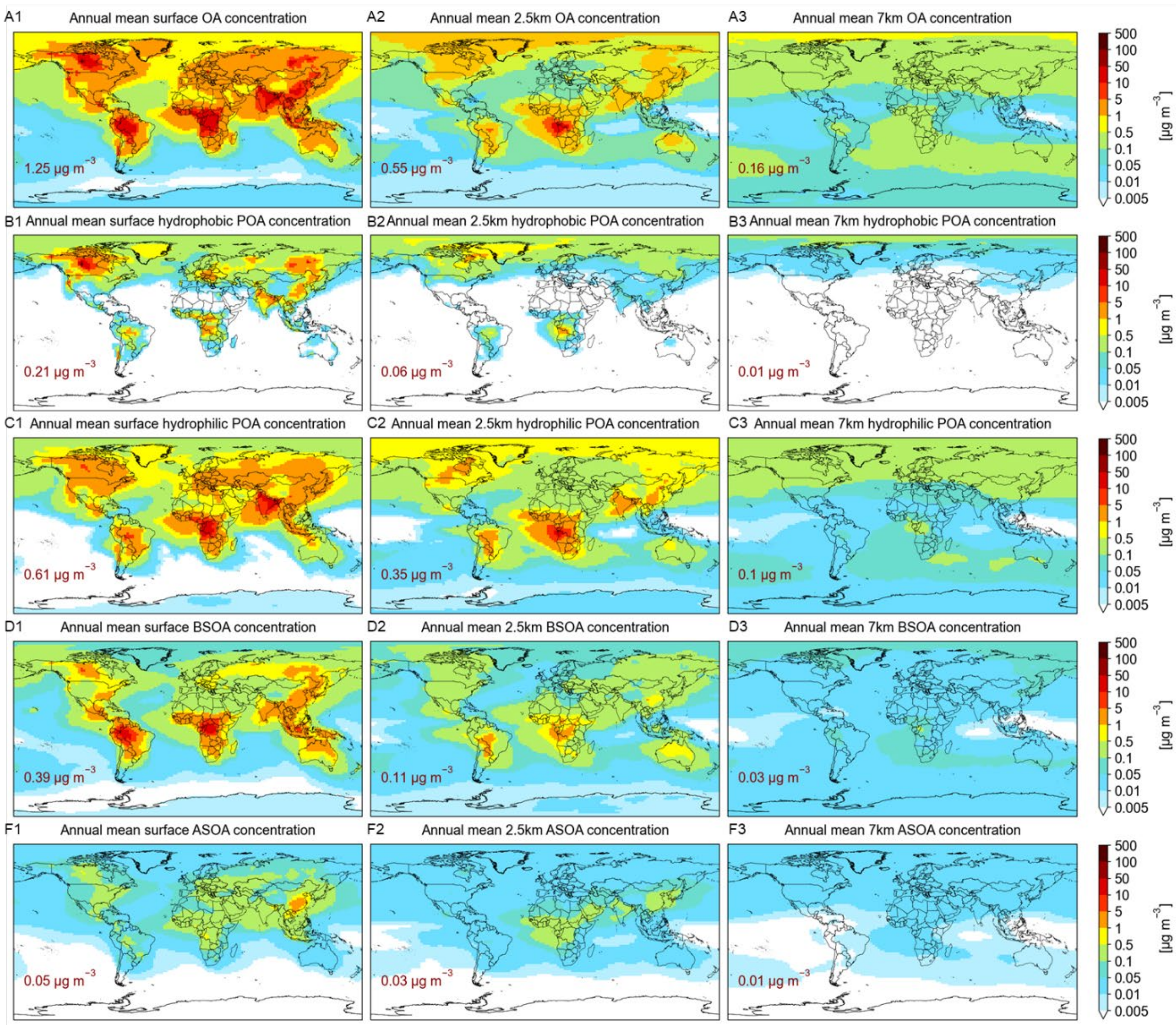

Figure S4. Simulated global annual (2017) mean source specific OA concentration at different attitudes. (A1-A3) total OA; (B1-B3) hydrophobic POA; (C1-C3) hydrophilic POA; (D1-D3) biogenic SOA; (F1-F3) anthropogenic SOA.

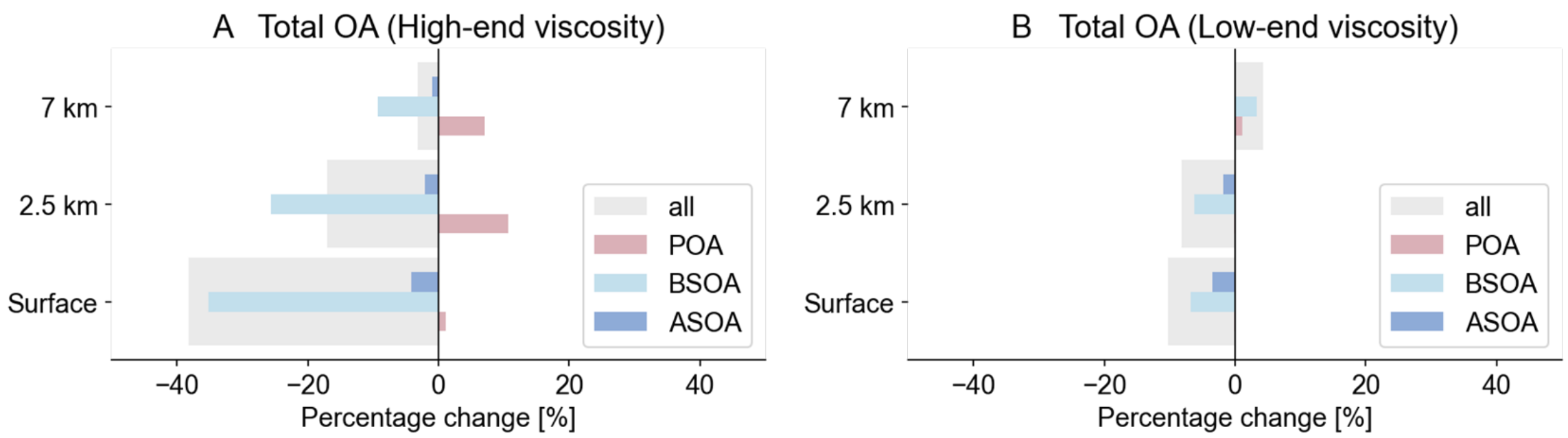

Figure S5. Sensitivity simulation mean source specific OA and total OA concentration change due to phase state effects at 3 specific altitudes under (A) high-end viscosity assumptions and (B) low-end viscosity assumptions.

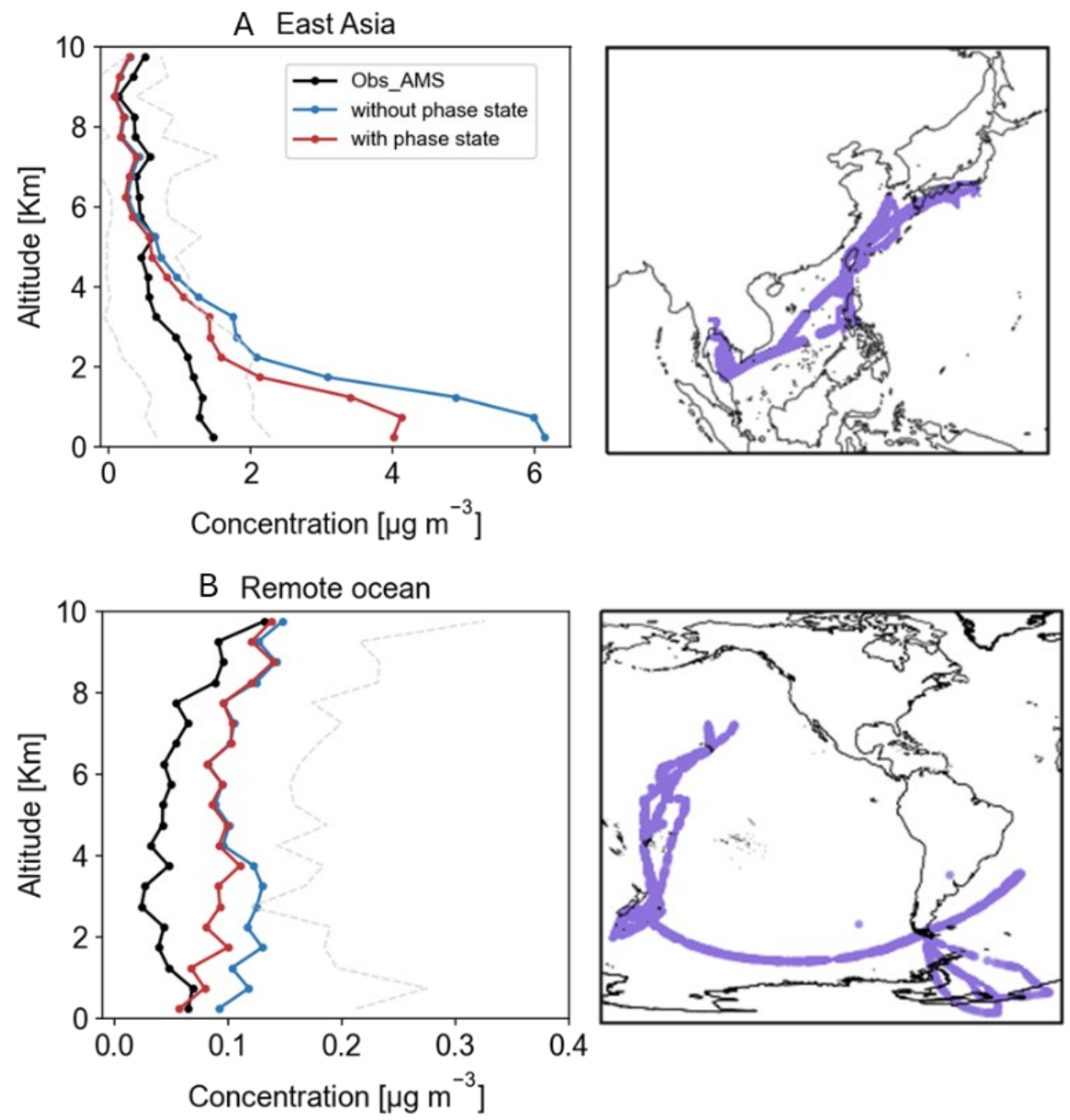

Figure S6. Comparison of simulated mean total OA vertical concentration profiles with and without phase state treatments against aircraft measurement data along with the aircraft maps. (A) East Asia, (B) Remote ocean.

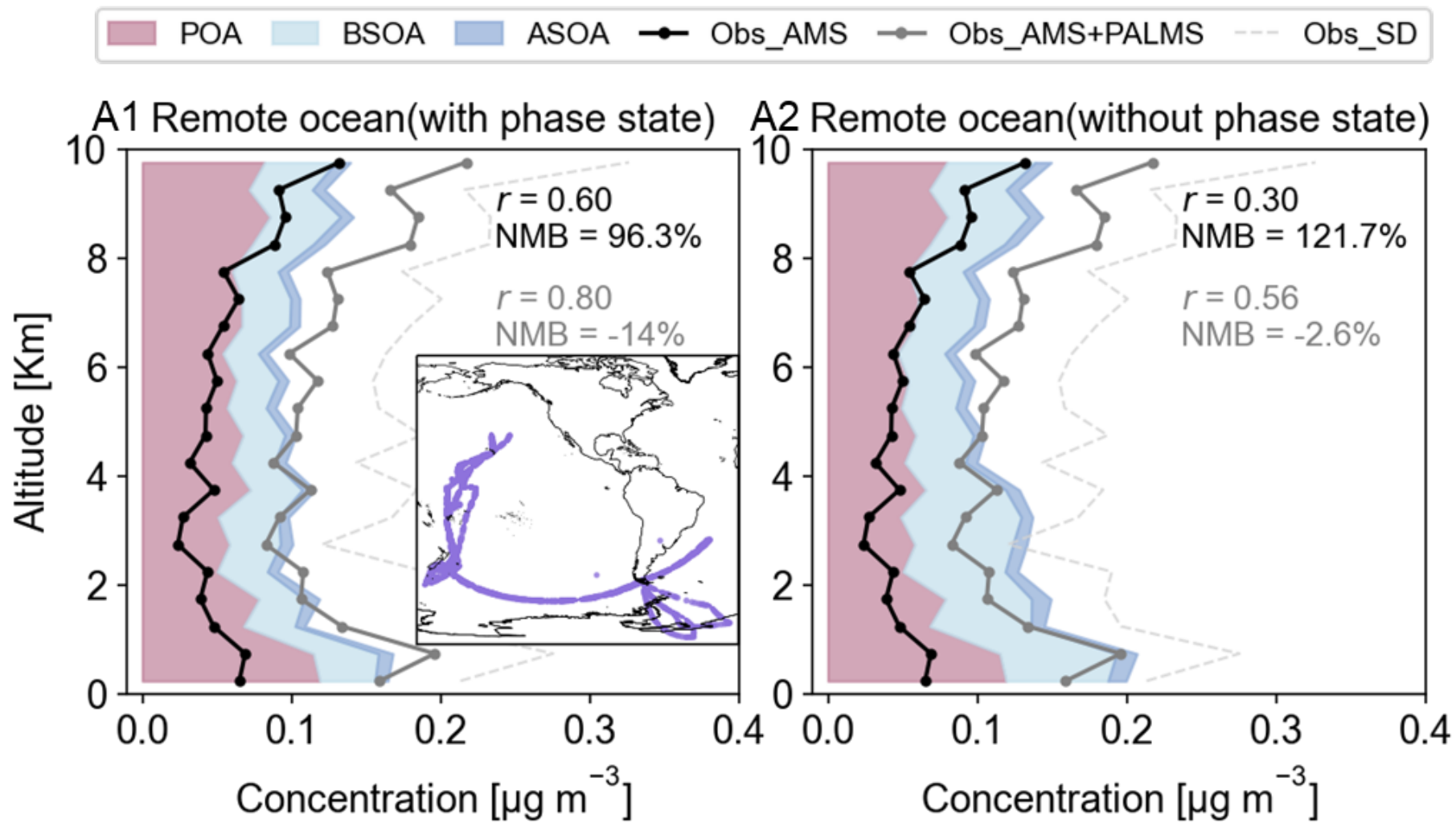

Figure S7. Comparison of simulated mean OA vertical concentration profiles with and without phase state treatments against aircraft measurement data. Simulated OA concentration profiles with (A1) and without (A2) phase state *vs* measurement from ATom1 to ATom4 campaign in remote ocean areas. The Pearson correlation coefficients (*r*) and normalized mean bias (NMB) are displayed in the inset. Obs_AMS indicate the AMS measured concentrations (black line), Obs_AMS+PALMS indicate the devided $PM_{2.5}$ OA concentrations from AMS and PALMS measurement (grey line) [1], and the Obs_SD indicate the standard deviation of the observations in each altitude bin. The aircraft flight tracks are also shown in the inset.

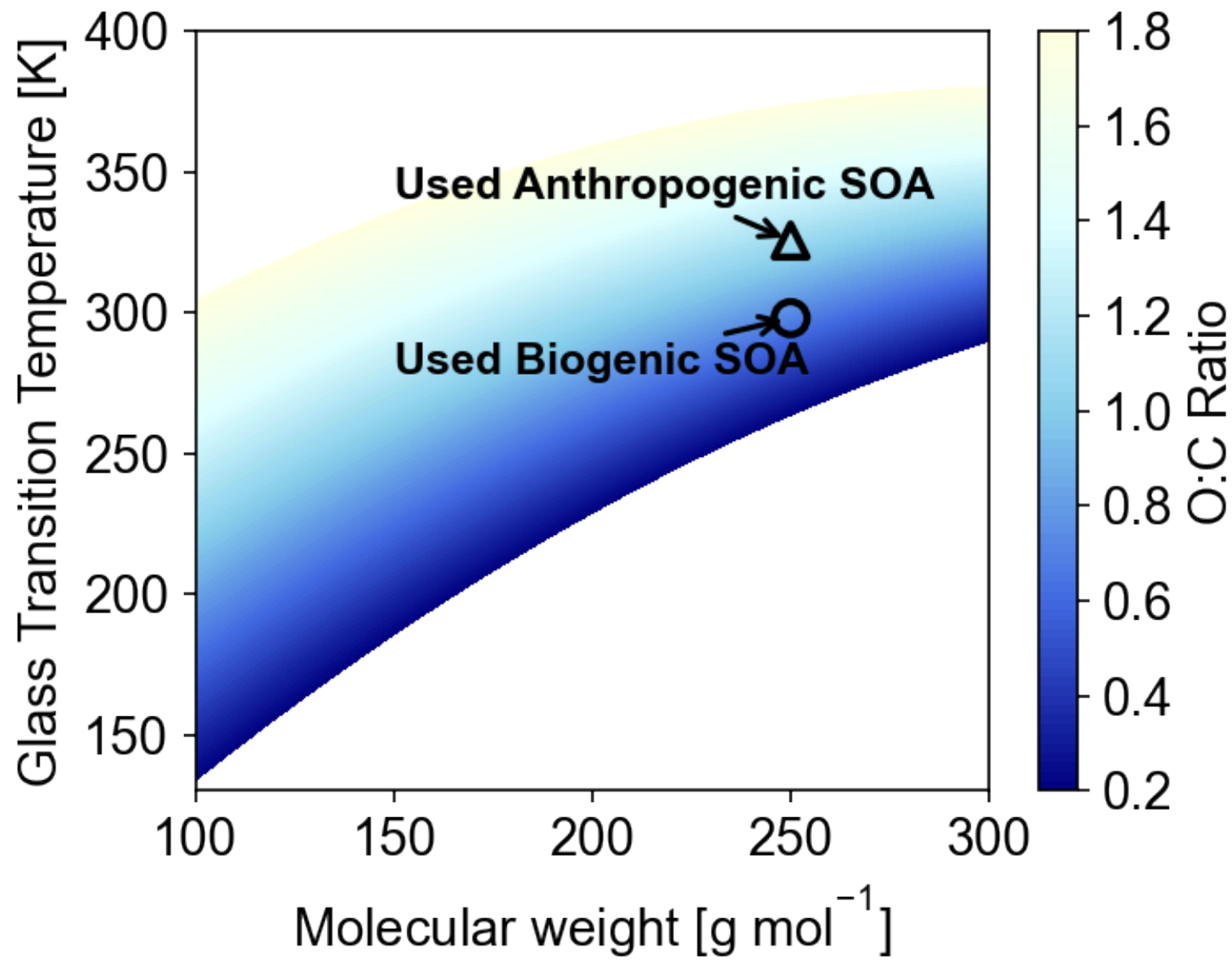

Figure S8. Predicted dry SOA T_g under different molecular weight and O:C ratio. The rang of molecular weight and O:C ratio get from Chen, et al. [2], and the T_g is calculated following Shiraiwa, et al. [3].

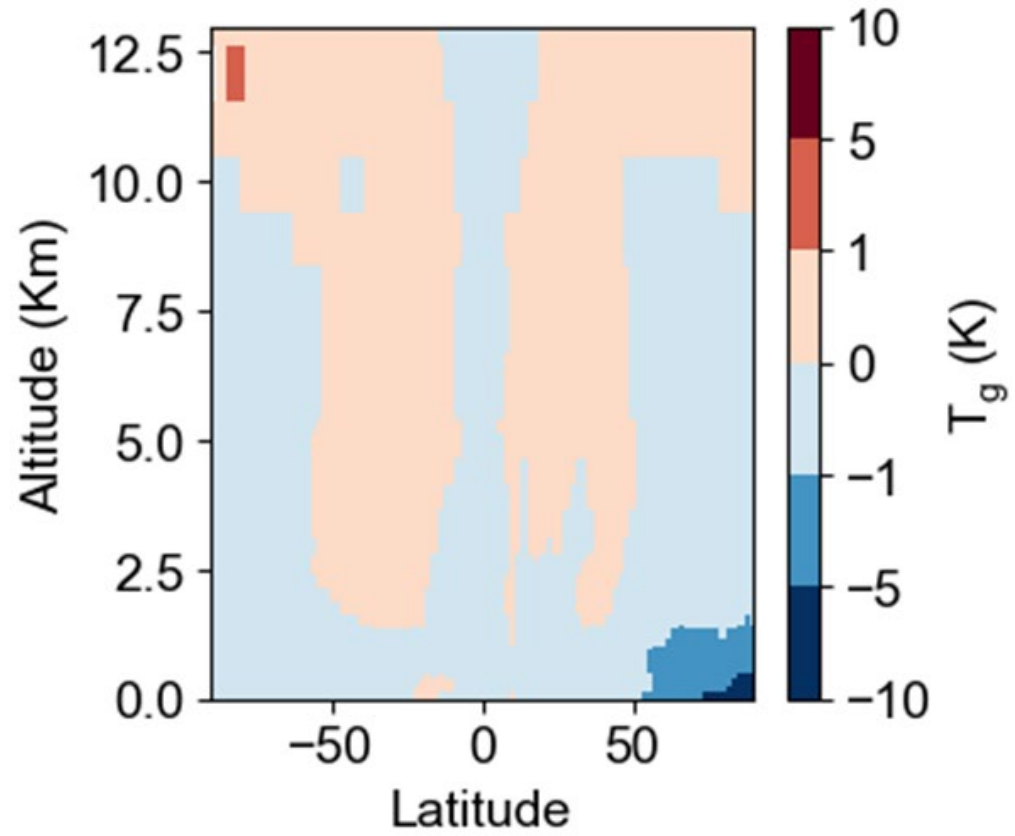

Figure S9. Sensitivity of predicted $T_{g,total}$ to external mixing calculations using dry versus hygroscopic-growth-adjusted OA mass weighting in Eq. 2.

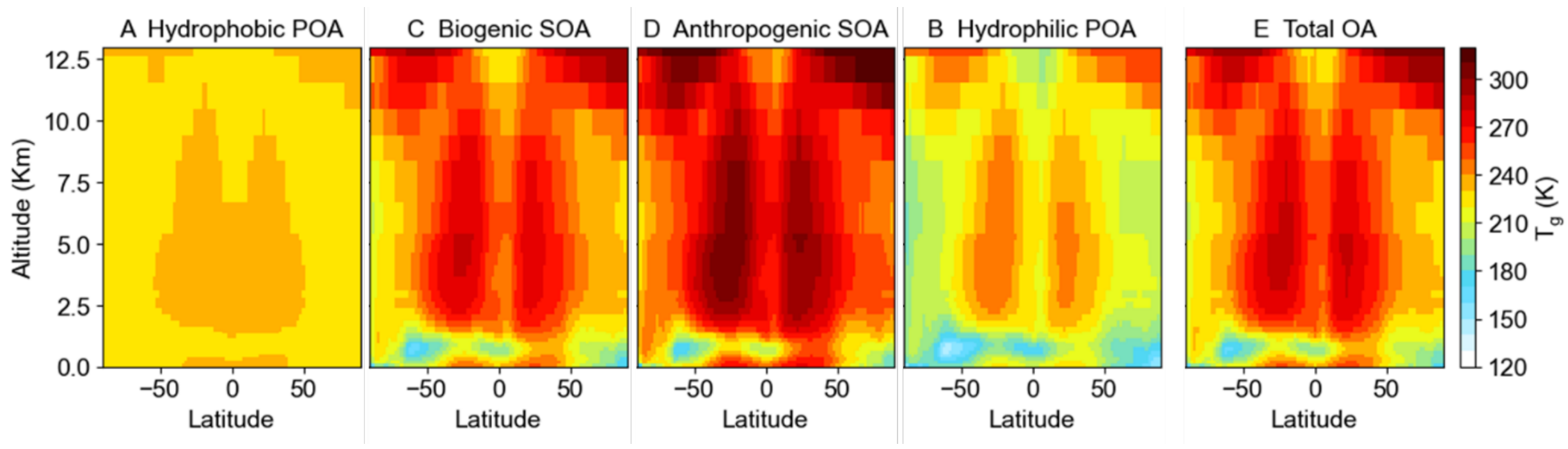

Figure S10. Simulated meridional mean source specific OA and total OA T_g. (A) hydrophilic POA, (B) hydrophobic POA, (C) biogenic SOA, (D) anthropogenic SOA, (E) total OA.

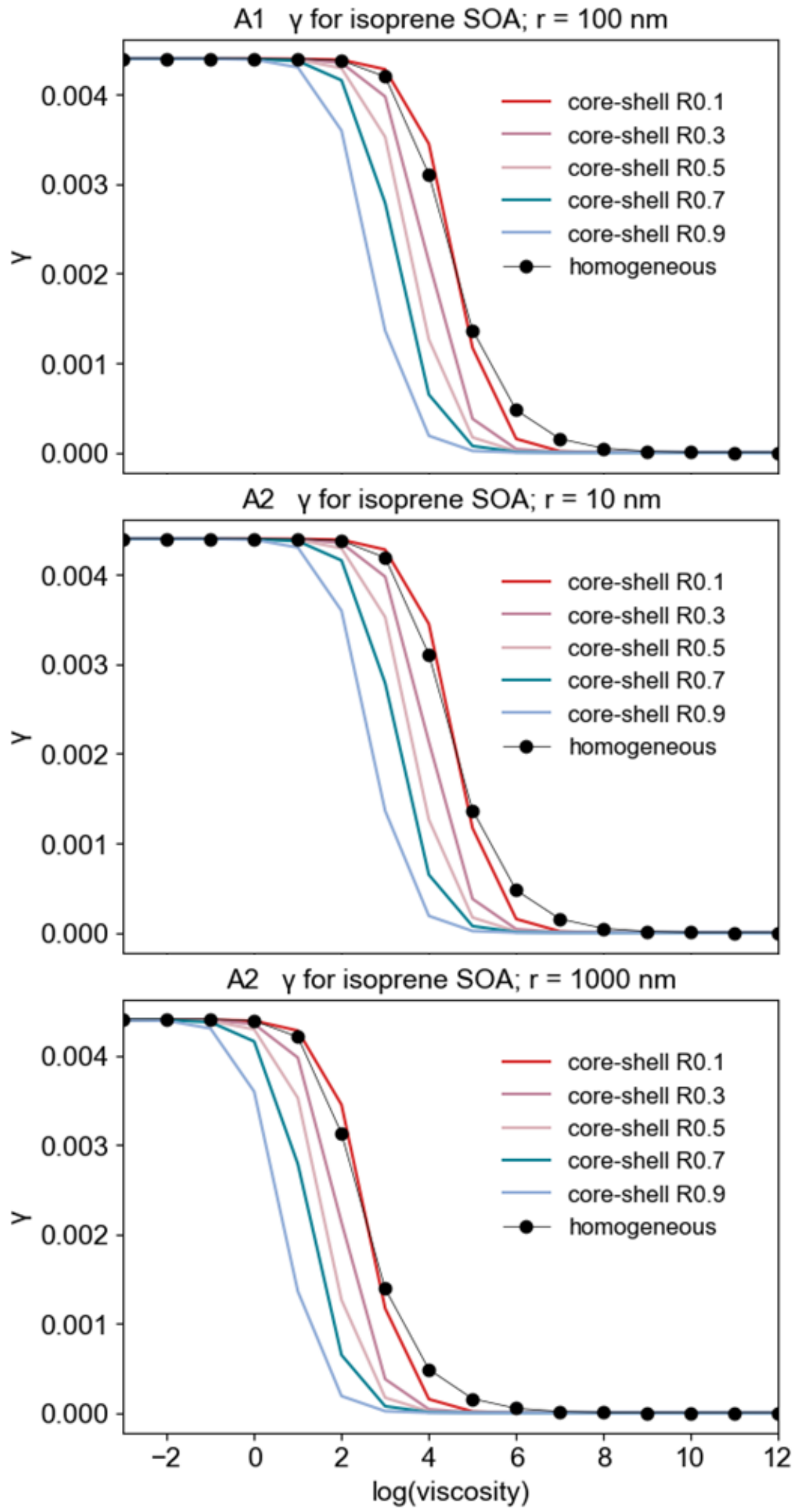

Figure S11. Predicted γ under different particle radius (*r*) and morphology assumptions, including homogeneous particles and core-shell particles with shell thicknesses ranging from 0.1 to 0.9 times of the particle radius. (A1) γ for isoprene SOA, *r* = 100 nm ; (A1) γ for isoprene SOA, *r* =10 nm ; (A1) γ for isoprene SOA, *r* = 1000 nm.

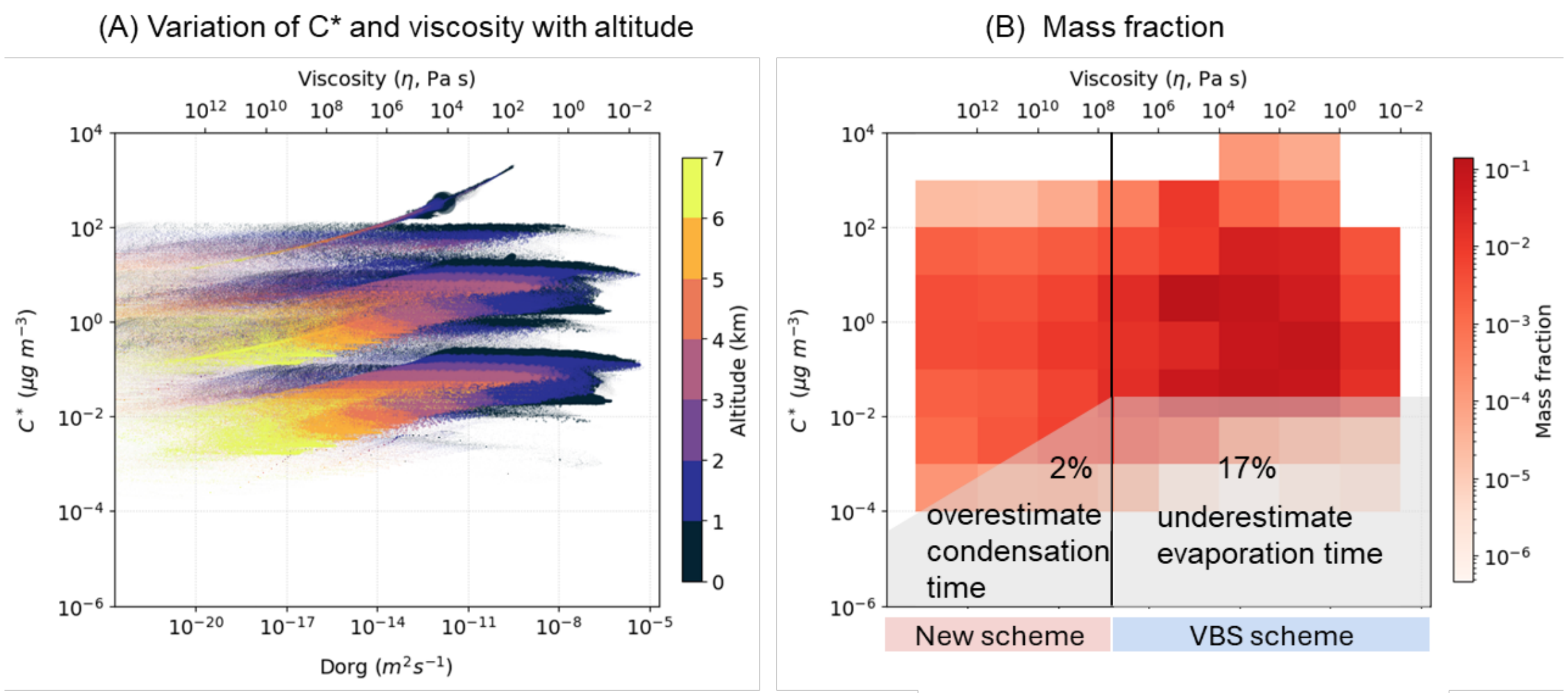

Figure S12. (A) Temperature-dependent C* and predicted viscosity across model grid boxes at different altitudes. (B) Simulated OA mass fraction under different temperature-dependent C* and viscosity conditions, along with the controlling scheme (New phase-state scheme or VBS scheme). Unshaded regions indicate OA ranges that can be represented by the corresponding schemes, while gray-shaded regions denote conditions that cannot be accurately captured for condensation or evaporation scenarios. 2% and 17% indicate fraction of simulated OA mass fraction in the respective gray-shaded regions.

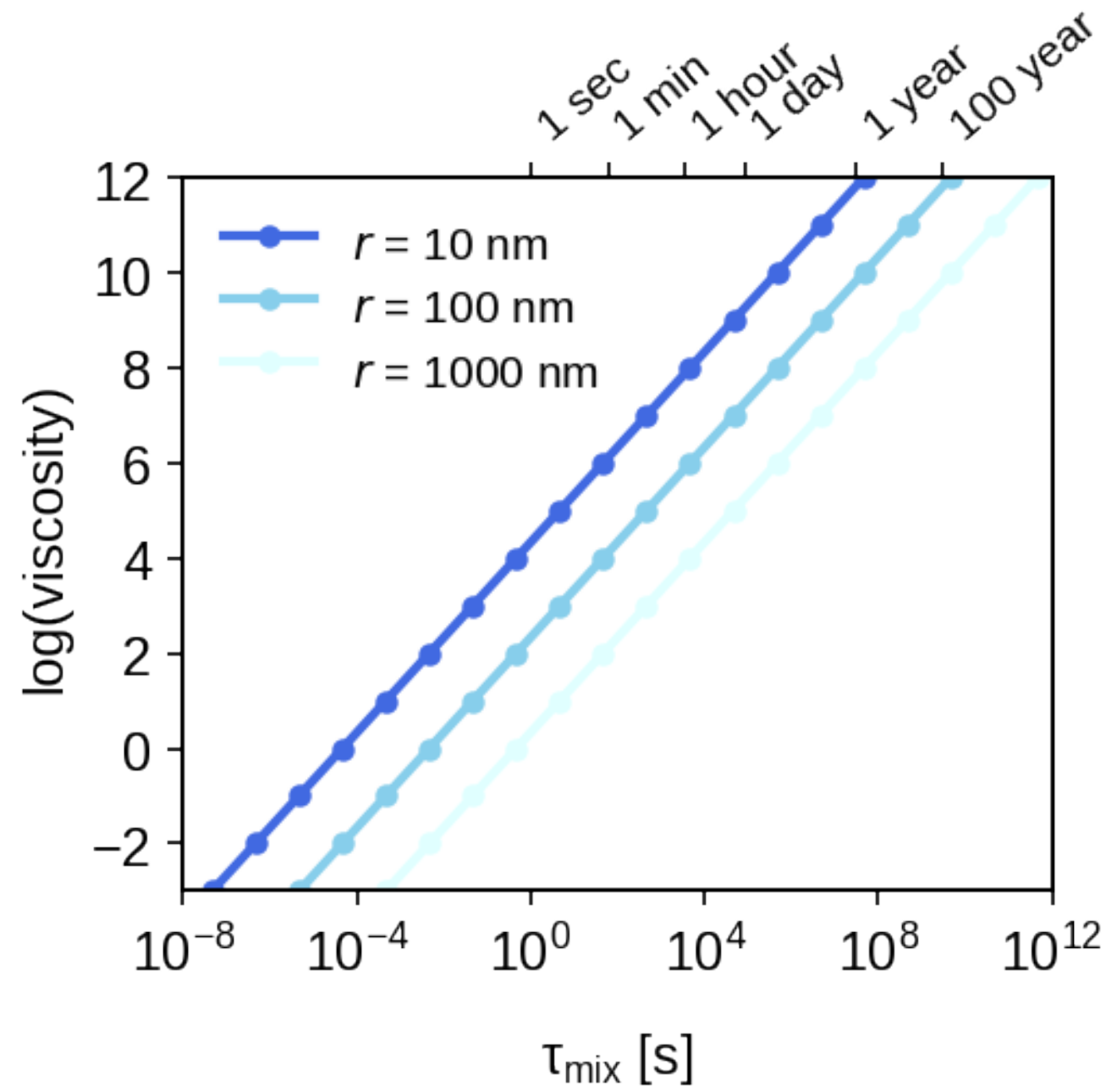

Figure S13. Predicted τ_{mix} change with viscosity under different particle radius asumptions (*r* =100 nm; *r*=10 nm; *r* =1000 nm).

Reference

1. Brock, C.; Froyd, K.; Dollner, M.; Williamson, C.; Schill, G.; Murphy, D.; Wagner, N.; Kupc, A.; Jimenez, J.; Campuzano-Jost, P.; Nault, B.; Schroder, J.; Day, D.; Price, D.; Weinzierl, B.; Schwarz, J.; Katich, J.; Wang, S.; Zeng, L.; Weber, R.; Dibb, J.; Scheuer, E.; Diskin, G.; DiGangi, J.; Bui, T.; Dean-Day, J.; Thompson, C.; Peischl, J.; Ryerson, T.; Bourgeois, I.; Daube, B.; Commane, R.; Wofsy, S. Ambient aerosol properties in the remote atmosphere from global-scale in situ measurements. *Atmospheric Chemistry and Physics* **2021**, *21* (19), 15023-15063. DOI: 10.5194/acp-21-15023-2021.

2. Chen, Q.; Heald, C.; Jimenez, J.; Canagaratna, M.; Zhang, Q.; He, L.; Huang, X.; Campuzano-Jost, P.; Palm, B.; Poulain, L.; Kuwata, M.; Martin, S.; Abbatt, J.; Lee, A.; Liggio, J. Elemental composition of organic aerosol: The gap between ambient and laboratory measurements. *Geophysical Research Letters* **2015**, *42* (10), 4182-4189. DOI: 10.1002/2015GL063693.

3. Shiraiwa, M.; Li, Y.; Tsimpidi, A. P.; Karydis, V. A.; Berkemeier, T.; Pandis, S. N.; Lelieveld, J.; Koop, T.; Poeschl, U. Global distribution of particle phase state in atmospheric secondary organic aerosols. *Nature Communications* **2017**, *8*, Article. DOI: 10.1038/ncomms15002.

4. Park, R. J.; Jacob, D. J.; Field, B. D.; Yantosca, R. M.; Chin, M. Natural and transboundary pollution influences on sulfate-nitrate-ammonium aerosols in the United States: Implications for policy. *Journal of Geophysical Research-Atmospheres* **2004**, *109* (D15), Article. DOI: 10.1029/2003jd004473.

5. Parrella, J. P.; Jacob, D. J.; Liang, Q.; Zhang, Y.; Mickley, L. J.; Miller, B.; Evans, M. J.; Yang, X.; Pyle, J. A.; Theys, N.; Van Roozendael, M. Tropospheric bromine chemistry: implications for present and pre-industrial ozone and mercury. *Atmospheric Chemistry and Physics* **2012**, *12* (15), 6723-6740. DOI: 10.5194/acp-12-6723-2012.

6. Mao, J. Q.; Paulot, F.; Jacob, D. J.; Cohen, R. C.; Crounse, J. D.; Wennberg, P. O.; Keller, C. A.; Hudman, R. C.; Barkley, M. P.; Horowitz, L. W. Ozone and organic nitrates over the eastern United States: Sensitivity to isoprene chemistry. *Journal of Geophysical Research-Atmospheres* **2013**, *118* (19), 11256-11268. DOI: 10.1002/jgrd.50817.

7. Park, R. J.; Jacob, D. J.; Chin, M.; Martin, R. V. Sources of carbonaceous aerosols over the United States and implications for natural visibility. *Journal of Geophysical Research-Atmospheres* **2003**, *108* (D12). DOI: 10.1029/2002jd003190.

8. Fairlie, T. D.; Jacob, D. J.; Park, R. J. The impact of transpacific transport of mineral dust in the United States. *Atmospheric Environment* **2007**, *41* (6), 1251-1266. DOI: 10.1016/j.atmosenv.2006.09.048.

9. Jaeglé, L.; Quinn, P. K.; Bates, T. S.; Alexander, B.; Lin, J. T. Global distribution of sea salt aerosols: new constraints from in situ and remote sensing observations. *Atmospheric Chemistry and Physics* **2011**, *11* (7), 3137-3157. DOI: 10.5194/acp-11-3137-2011.

10. Pai, S. J.; Heald, C. L.; Pierce, J. R.; Farina, S. C.; Marais, E. A.; Jimenez, J. L.; Campuzano-Jost, P.; Nault, B. A.; Middlebrook, A. M.; Coe, H.; Shilling, J. E.; Bahreini, R.; Dingle, J. H.; Vu, K. An evaluation of global organic aerosol schemes using airborne observations. *Atmospheric Chemistry and Physics* **2020**, *20* (5), 2637-2665. DOI: 10.5194/acp-20-2637-2020.

11. Pye, H. O. T.; Seinfeld, J. H. A global perspective on aerosol from low-volatility organic compounds. *Atmospheric Chemistry and Physics* **2010**, *10* (9), 4377-4401. DOI: 10.5194/acp-10-4377-2010.

12. Marais, E. A.; Jacob, D. J.; Jimenez, J. L.; Campuzano-Jost, P.; Day, D. A.; Hu, W.; Krechmer, J.; Zhu, L.; Kim, P. S.; Miller, C. C.; Fisher, J. A.; Travis, K.; Yu, K.; Hanisco, T. F.; Wolfe, G. M.; Arkinson, H. L.; Pye, H. O. T.; Froyd, K. D.; Liao, J.; McNeill, V. F. Aqueous-phase mechanism for secondary organic aerosol formation from isoprene: application to the southeast United States and co-benefit of SO2 emission controls. *Atmospheric Chemistry and Physics* **2016**, *16* (3), 1603-1618. DOI: 10.5194/acp-16-1603-2016.

13. Bates, K. H.; Jacob, D. J. A new model mechanism for atmospheric oxidation of isoprene: global effects on oxidants, nitrogen oxides, organic products, and secondary organic aerosol. *Atmospheric Chemistry and Physics* **2019**, *19* (14), 9613-9640. DOI: 10.5194/acp-19-9613-2019.

14. Fisher, J.; Jacob, D.; Travis, K.; Kim, P.; Marais, E.; Miller, C.; Yu, K.; Zhu, L.; Yantosca, R.; Sulprizio, M.; Mao, J.; Wennberg, P.; Crounse, J.; Teng, A.; Nguyen, T.; St Clair, J.; Cohen, R.; Romer, P.; Nault, B.; Wooldridge, P.; Jimenez, J.; Campuzano-Jost, P.; Day, D.; Hu, W.; Shepson, P.; Xiong, F.; Blake, D.; Goldstein, A.; Misztal, P.; Hanisco, T.; Wolfe, G.; Ryerson, T.; Wisthaler, A.; Mikoviny, T. Organic nitrate chemistry and its implications for nitrogen budgets in an isoprene- and monoterpene-rich atmosphere: constraints from aircraft (SEAC4RS) and ground-based (SOAS) observations in the Southeast US. *Atmospheric Chemistry and Physics* **2016**, *16* (9), 5969-5991. DOI: 10.5194/acp-16-5969-2016.

15. Bates, K.; Jacob, D.; Li, K.; Ivatt, P.; Evans, M.; Yan, Y.; Lin, J. Development and evaluation of a new compact mechanism for aromatic oxidation in atmospheric models. *Atmospheric Chemistry and Physics* **2021**, *21* (24), 18351-18374. DOI: 10.5194/acp-21-18351-2021.

16. Fu, T.-M.; Jacob, D. J.; Wittrock, F.; Burrows, J. P.; Vrekoussis, M.; Henze, D. K. Global budgets of atmospheric glyoxal and methylglyoxal, and implications for formation of secondary organic aerosols. *Journal of Geophysical Research-Atmospheres* **2008**, *113* (D15). DOI: 10.1029/2007jd009505.

17. Lin, S. J.; Rood, R. B. Multidimensional flux-form semi-Lagrangian transport schemes. *Monthly Weather Review* **1996**, *124* (9), 2046-2070. DOI: 10.1175/1520-0493(1996)124<2046:mffslt>2.0.co;2.

18. Wesely, M. L. Parameterization of surface resistances to gaseous dry deposition in regional-scale numerical models. *Atmospheric Environment* **1989**, *23* (6), 1293-1304. DOI: 10.1016/0004-6981(89)90153-4.

19. Zhang, L. M.; Gong, S. L.; Padro, J.; Barrie, L. A size-segregated particle dry deposition scheme for an atmospheric aerosol module. *Atmospheric Environment* **2001**, *35* (3), 549-560. DOI: 10.1016/s1352-2310(00)00326-5.

20. Liu, H. Y.; Jacob, D. J.; Bey, I.; Yantosca, R. M. Constraints from Pb-210 and Be-7 on wet deposition and transport in a global three-dimensional chemical tracer model driven by assimilated meteorological fields. *Journal of Geophysical Research-Atmospheres* **2001**, *106* (D11), 12109-12128. DOI: 10.1029/2000jd900839.

21. Amos, H. M.; Jacob, D. J.; Holmes, C. D.; Fisher, J. A.; Wang, Q.; Yantosca, R. M.; Corbitt, E. S.; Galarneau, E.; Rutter, A. P.; Gustin, M. S.; Steffen, A.; Schauer, J. J.; Graydon, J. A.; St Louis, V. L.; Talbot, R. W.; Edgerton, E. S.; Zhang, Y.; Sunderland, E. M. Gas-particle partitioning of atmospheric Hg(II) and its effect on global mercury deposition. *Atmospheric Chemistry and Physics* **2012**, *12* (1), 591-603. DOI: 10.5194/acp-12-591-2012.

22. McDuffie, E. E.; Smith, S. J.; O'Rourke, P.; Tibrewal, K.; Venkataraman, C.; Marais, E. A.; Zheng, B.; Crippa, M.; Brauer, M.; Martin, R. V. A global anthropogenic emission inventory of atmospheric pollutants from sector- and fuel-specific sources (1970-2017): an application of the Community Emissions Data System (CEDS). *Earth System Science Data* **2020**, *12* (4), 3413-3442, Article; Data Paper. DOI: 10.5194/essd-12-3413-2020.

23. Marais, E. A.; Wiedinmyer, C. Air quality impact of diffuse and inefficient combustion emissions in Africa (DICE-Africa). *Environmental Science & Technology* **2016**, *50* (19), 10739-10745. DOI: 10.1021/acs.est.6b02602.

24. Zheng, B.; Tong, D.; Li, M.; Liu, F.; Hong, C.; Geng, G.; Li, H.; Li, X.; Peng, L.; Qi, J.; Yan, L.; Zhang, Y.; Zhao, H.; Zheng, Y.; He, K.; Zhang, Q. Trends in China's anthropogenic emissions since 2010 as the consequence of clean air actions. *Atmospheric Chemistry and Physics* **2018**, *18* (19), 14095-14111, Article. DOI: 10.5194/acp-18-14095-2018.

25. van der Werf, G. R.; Randerson, J. T.; Giglio, L.; van Leeuwen, T. T.; Chen, Y.; Rogers, B. M.; Mu, M. Q.; van Marle, M. J. E.; Morton, D. C.; Collatz, G. J.; Yokelson, R. J.; Kasibhatla, P. S. Global fire emissions estimates during 1997-2016. *Earth System Science Data* **2017**, *9* (2), 697-720, Article. DOI: 10.5194/essd-9-697-2017.

26. Guenther, A. B.; Jiang, X.; Heald, C. L.; Sakulyanontvittaya, T.; Duhl, T.; Emmons, L. K.; Wang, X. The model of rmissions of gases and aerosols from Nature version 2.1 (MEGAN2.1): an extended and updated framework for modeling biogenic emissions. *Geoscientific Model Development* **2012**, *5* (6), 1471-1492. DOI: 10.5194/gmd-5-1471-2012.

27. Gregson, F. K. A.; Gerrebos, N. G. A.; Schervish, M.; Nikkho, S.; Schnitzler, E. G.; Schwartz, C.; Carlsten, C.; Abbatt, J. P. D.; Kamal, S.; Shiraiwa, M.; Bertram, A. K. Phase Behavior and Viscosity in Biomass Burning Organic Aerosol and Climatic Impacts. *Environmental Science & Technology* **2023**, *57* (39), 14548-14557. DOI: 10.1021/acs.est.3c03231.

28. Schnitzler, E. G.; Liu, T.; Hems, R. F.; Abbatt, J. P. D. Emerging investigator series: heterogeneous OH oxidation of primary brown carbon aerosol: effects of relative humidity and volatility. *Environmental Science-Processes & Impacts* **2020**, *22* (11), 2162-2171. DOI: 10.1039/d0em00311e.

29. Xu, W.; Li, Z.; Zhang, Z.; Li, J.; Karnezi, E.; Lambe, A.; Zhou, W.; Sun, J.; Du, A.; Li, Y.; Sun, Y. Changes in Physicochemical Properties of Organic Aerosol During Photochemical Aging of Cooking and Burning Emissions. *Journal of Geophysical Research-Atmospheres* **2023**, *128* (14). DOI: 10.1029/2022JD037911.

30. Gerrebos, N.; Zaks, J.; Gregson, F.; Walton-Raaby, M.; Meeres, H.; Zigg, I.; Zandberg, W.; Bertram, A. High Viscosity and Two Phases Observed over a Range of Relative Humidities in Biomass Burning Organic Aerosol from Canadian Wildfires. *Environmental Science & Technology* **2024**, *58* (49), 21716-21728. DOI: 10.1021/acs.est.4c09148.

31. Siemens, K.; Paik, T.; Li, A.; Rivera-Adorno, F.; Tomlin, J.; Xie, Q.; Chakrabarty, R.; Laskin, A. Light Absorption and Chemical Composition of Brown Carbon Organic Aerosol Produced from Burning of Selected Biofuels. *Acs Earth and Space Chemistry* **2024**, *8* (7), 1416-1428. DOI: 10.1021/acsearthspacechem.4c00056.

32. Mahrt, F.; Huang, Y.; Zaks, J.; Devi, A.; Peng, L.; Ohno, P.; Qin, Y.; Martin, S.; Ammann, M.; Bertram, A. Phase Behavior of Internal Mixtures of Hydrocarbon-like Primary Organic Aerosol and Secondary Aerosol Based on Their Differences in Oxygen-to-Carbon Ratios. *Environmental Science & Technology* **2022**, *56* (7), 3960-3973. DOI: 10.1021/acs.est.1c07691.

33. DeRieux, W. S.; Li, Y.; Lin, P.; Laskin, J.; Laskin, A.; Bertram, A. K.; Nizkorodov, S. A.; Shiraiwa, M. Predicting the glass transition temperature and viscosity of secondary organic material using molecular composition. *Atmospheric Chemistry and Physics* **2018**, *18* (9), 6331-6351. DOI: 10.5194/acp-18-6331-2018.

34. Li, Y.; Day, D. A.; Stark, H.; Jimenez, J. L.; Shiraiwa, M. Predictions of the glass transition temperature and viscosity of organic aerosols from volatility distributions. *Atmospheric Chemistry and Physics* **2020**, *20* (13), 8103-8122. DOI: 10.5194/acp-20-8103-2020.

35. Zhang, Y.; Nichman, L.; Spencer, P.; Jung, J.; Lee, A.; Heffernan, B.; Gold, A.; Zhang, Z.; Chen, Y.; Canagaratna, M.; Jayne, J.; Worsnop, D.; Onasch, T.; Surratt, J.; Chandler, D.; Davidovits, P.; Kolb, C. The Cooling Rate- and Volatility-Dependent Glass-Forming Properties of Organic Aerosols Measured by Broadband Dielectric Spectroscopy. *Environmental Science & Technology* **2019**, *53* (21), 12366-12378. DOI: 10.1021/acs.est.9b03317.

36. Koop, T.; Bookhold, J.; Shiraiwa, M.; Pöschl, U. Glass transition and phase state of organic compounds: dependency on molecular properties and implications for secondary organic aerosols in the atmosphere. *Physical Chemistry Chemical Physics* **2011**, *13* (43), 19238-19255. DOI: 10.1039/c1cp22617g.

37. Reid, J. P.; Bertram, A. K.; Topping, D. O.; Laskin, A.; Martin, S. T.; Petters, M. D.; Pope, F. D.; Rovelli, G. The viscosity of atmospherically relevant organic particles. *Nature Communications* **2018**, *9*. DOI: 10.1038/s41467-018-03027-z.

38. Zobrist, B.; Marcolli, C.; Pedernera, D.; Koop, T. Do atmospheric aerosols form glasses? *Atmospheric Chemistry and Physics* **2008**, *8* (17), 5221-5244.

39. Petters, M.; Kreidenweis, S. A single parameter representation of hygroscopic growth and cloud condensation nucleus activity - Part 3: Including surfactant partitioning. *Atmospheric Chemistry and Physics* **2013**, *13* (2), 1081-1091. DOI: 10.5194/acp-13-1081-2013.

40. Kuwata, M.; Zorn, S.; Martin, S. Using Elemental Ratios to Predict the Density of Organic Material Composed of Carbon, Hydrogen, and Oxygen. *Environmental Science & Technology* **2012**, *46* (2), 787-794. DOI: 10.1021/es202525q.

41. Kostenidou, E.; Pathak, R.; Pandis, S. An algorithm for the calculation of secondary organic aerosol density combining AMS and SMPS data. *Aerosol Science and Technology* **2007**, *41* (11), 1002-1010. DOI: 10.1080/02786820701666270.

42. Cross, E.; Slowik, J.; Davidovits, P.; Allan, J.; Worsnop, D.; Jayne, J.; Lewis, D.; Canagaratna, M.; Onasch, T. Laboratory and ambient particle density determinations using light scattering in conjunction with aerosol mass spectrometry. *Aerosol Science and Technology* **2007**, *41* (4), 343-359. DOI: 10.1080/02786820701199736.

43. Wu, Z.; Poulain, L.; Henning, S.; Dieckmann, K.; Birmili, W.; Merkel, M.; van Pinxteren, D.; Spindler, G.; Müller, K.; Stratmann, F.; Herrmann, H.; Wiedensohler, A. Relating particle hygroscopicity and CCN activity to chemical composition during the HCCT-2010 field campaign. *Atmospheric Chemistry and Physics* **2013**, *13* (16), 7983-7996. DOI: 10.5194/acp-13-7983-2013.

44. Hildebrandt Ruiz, L.; Paciga, A.; Cerully, K.; Nenes, A.; Donahue, N.; Pandis, S. Formation and aging of secondary organic aerosol from toluene: changes in chemical composition, volatility, and hygroscopicity. *Atmospheric Chemistry and Physics* **2015**, *15* (14), 8301-8313. DOI: 10.5194/acp-15-8301-2015.

45. Frosch, M.; Bilde, M.; Nenes, A.; Praplan, A.; Jurányi, Z.; Dommen, J.; Gysel, M.; Weingartner, E.; Baltensperger, U. CCN activity and volatility of β-caryophyllene secondary organic aerosol. *Atmospheric Chemistry and Physics* **2013**, *13* (4), 2283-2297. DOI: 10.5194/acp-13-2283-2013.

46. Luu, R.; Schervish, M.; June, N.; O'Donnell, S.; Jathar, S.; Pierce, J.; Shiraiwa, M. Global Simulations of Phase State and Equilibration Time Scales of Secondary Organic Aerosols with GEOS-Chem. *Acs Earth and Space Chemistry* **2025**, *9* (2), 288-302. DOI: 10.1021/acsearthspacechem.4c00281.

47. Zhang, Z.; Li, Y.; Ran, H.; An, J.; Qu, Y.; Zhou, W.; Xu, W.; Hu, W.; Xie, H.; Wang, Z.; Sun, Y.; Shiraiwa, M. Simulated phase state and viscosity of secondary organic aerosols over China. *Atmospheric Chemistry and Physics* **2024**, *24*, 4809-4826. DOI: 10.5194/acp-24-4809-2024.

48. Wang, Y.; Voliotis, A.; Shao, Y. Q.; Zong, T. M.; Meng, X. X. Y.; Du, M.; Hu, D. W.; Chen, Y.; Wu, Z. J.; Alfarra, M. R.; McFiggans, G. Phase state of secondary organic aerosol in chamber photo-oxidation of mixed precursors. *Atmospheric Chemistry and Physics* **2021**, *21* (14), 11303-11316. DOI: 10.5194/acp-21-11303-2021.

49. Lei, Z.; Chen, Y.; Zhang, Y.; Cooke, M.; Ledsky, I.; Armstrong, N.; Olson, N.; Zhang, Z.; Gold, A.; Surratt, J.; Ault, A. Initial pH Governs Secondary Organic Aerosol Phase State and Morphology after Uptake of Isoprene Epoxydiols (IEPDX). *Environmental Science & Technology* **2022**, *56* (15), 10596-10607. DOI: 10.1021/acs.est.2c01579.

50. Gorkowski, K.; Donahue, N.; Sullivan, R. Aerosol Optical Tweezers Constrain the Morphology Evolution of Liquid-Liquid Phase-Separated Atmospheric Particles. *CHEM* **2020**, *6* (1), 204-220. DOI: 10.1016/j.chempr.2019.10.018.

51. Ye, Y.; Sun, J.; Fan, Y.; Li, Y.; Li, Q.; He, C.; Ma, S.; Zhao, Z.; Xu, T. Key Roles of Bulk Viscosity and Acidity on Liquid-Liquid Phase Separation of Atmospheric Organic-Inorganic Mixed Aerosols. *Journal of Physical Chemistry A* **2025**, *129* (17), 3921-3930. DOI: 10.1021/acs.jpca.5c01182.

52. You, Y.; Smith, M.; Song, M.; Martin, S.; Bertram, A. Liquid-liquid phase separation in atmospherically relevant particles consisting of organic species and inorganic salts. *INTERNATIONAL REVIEWS IN PHYSICAL CHEMISTRY* **2014**, *33* (1), 43-77. DOI: 10.1080/0144235X.2014.890786.

53. Davidovits, P.; Kolb, C.; Williams, L.; Jayne, J.; Worsnop, D. Mass accommodation and chemical reactions at gas-liquid interfaces. *Chemical Reviews* **2006**, *106*, 1323-1354. DOI: 10.1021/cr040366k.

54. Kolb, C.; Cox, R.; Abbatt, J.; Ammann, M.; Davis, E.; Donaldson, D.; Garrett, B.; George, C.; Griffiths, P.; Hanson, D.; Kulmala, M.; McFiggans, G.; Pöschl, U.; Riipinen, I.; Rossi, M.; Rudich, Y.; Wagner, P.; Winkler, P.; Worsnop, D.; O'Dowd, C. An overview of current issues in the uptake of atmospheric trace gases by aerosols and clouds. *Atmospheric Chemistry and Physics* **2010**, *10* (21), 10561-10605. DOI: 10.5194/acp-10-10561-2010.

55. Anttila, T.; Kiendler-Scharr, A.; Tillmann, R.; Mentel, T. On the reactive uptake of gaseous compounds by organic-coated aqueous aerosols:: Theoretical analysis and application to the heterogeneous hydrolysis of N2O5. *Journal of Physical Chemistry A* **2006**, *110* (35), 10435-10443. DOI: 10.1021/jp062403c.

56. Zhang, Y.; Chen, Y. Z.; Lambe, A. T.; Olson, N. E.; Lei, Z. Y.; Craig, R. L.; Zhang, Z. F.; Gold, A.; Onasch, T. B.; Jayne, J. T.; Worsnop, D. R.; Gaston, C. J.; Thornton, J. A.; Vizuete, W.; Ault, A. P.; Surratt, J. D. Effect of the Aerosol-Phase State on Secondary Organic Aerosol Formation from the Reactive Uptake of Isoprene-Derived Epoxydiols (IEPDX). *Environmental Science & Technology Letters* **2018**, *5* (3), 167-174. DOI: 10.1021/acs.estlett.8b00044.

57. Pankow, J. An absorption model of the gas/aerosol partitioning involved in the formation of secondary organic aerosol. *Atmosphere Environment* **1994**, *28* (2), 189-193, Article. DOI: 10.1016/1352-2310(94)90094-9.

58. Li, Y.; Shiraiwa, M. Timescales of secondary organic aerosols to reach equilibrium at various temperatures and relative humidities. *Atmospheric Chemistry and Physics* **2019**, *19* (9), 5959-5971. DOI: 10.5194/acp-19-5959-2019.

59. Kim, D.; Campuzano-Jost, P.; Guo, H.; Day, D. A.; Yang, D.; Dhaniyala, S.; Williams, L.; Croteau, P.; Jayne, J.; Worsnop, D.; Volkamer, R.; Jimenez, J. L. Development and characterization of an aircraft inlet system for broader quantitative particle sampling at higher altitudes: aerodynamic lenses, beam and vaporizer diagnostics, and pressure-controlled inlets. *Aerosol Research* **2025**, *3* (2), 371-404. DOI: 10.5194/ar-3-371-2025.

60. Guo, H.; Campuzano-Jost, P.; Nault, B.; Day, D.; Schroder, J.; Kim, D.; Dibb, J.; Dollner, M.; Weinzierl, B.; Jimenez, J. The importance of size ranges in aerosol instrument intercomparisons: a case study for the Atmospheric Tomography Mission. *Atmospheric Measurement Techniques* **2021**, *14* (5), 3631-3655. DOI: 10.5194/amt-14-3631-2021.

61. Brock, C.; Williamson, C.; Kupc, A.; Froyd, K.; Erdesz, F.; Wagner, N.; Richardson, M.; Schwarz, J.; Gao, R.; Katich, J.; Campuzano-Jost, P.; Nault, B.; Schroder, J.; Jimenez, J.; Weinzierl, B.; Dollner, M.; Bui, T.; Murphy, D. Aerosol size distributions during the Atmospheric Tomography Mission (ATom): methods, uncertainties, and data products. *Atmospheric Measurement Techniques* **2019**, *12* (6), 3081-3099. DOI: 10.5194/amt-12-3081-2019.

62. Shiraiwa, M.; Seinfeld, J. H. Equilibration timescale of atmospheric secondary organic aerosol partitioning. *Geophysical Research Letters* **2012**, *39*. DOI: 10.1029/2012gl054008.

63. Zaveri, R. A.; Easter, R. C.; Shilling, J. E.; Seinfeld, J. H. Modeling kinetic partitioning of secondary organic aerosol and size distribution dynamics: representing effects of volatility, phase state, and particle-phase reaction. *Atmospheric Chemistry and Physics* **2014**, *14* (10), 5153-5181. DOI: 10.5194/acp-14-5153-2014.
